# Sublimable chloroquinolinate lanthanoid single-ion magnets deposited on ferromagnetic electrodes†
†Electronic supplementary information (ESI) available. CCDC 1557647–1557649. For ESI and crystallographic data in CIF or other electronic format see DOI: 10.1039/c7sc03463f


**DOI:** 10.1039/c7sc03463f

**Published:** 2017-10-17

**Authors:** Sara G. Miralles, Amilcar Bedoya-Pinto, José J. Baldoví, Walter Cañon-Mancisidor, Yoann Prado, Helena Prima-Garcia, Alejandro Gaita-Ariño, Guillermo Mínguez Espallargas, Luis E. Hueso, Eugenio Coronado

**Affiliations:** a Instituto de Ciencia Molecular (ICMol) , Universidad de Valencia , Catedrático José Beltrán 2, 46980 Paterna , Valencia , Spain . Email: eugenio.coronado@uv.es; b CIC nanoGUNE , Tolosa Hiribidea 76 , 20018 Donostia-San Sebastián , Spain . Email: l.hueso@nanogune.eu; c Max-Planck Institute of Microstructure Physics , Weinberg 2 , 06120 Halle , Germany; d Max Planck Institute for the Structure and Dynamics of Matter , Luruper Chaussee 149 , D-22761 Hamburg , Germany; e Facultad de Química y Biologia , Depto. de Química de los Materiales , Universidad de Santiago de Chile , USACH , Chile

## Abstract

A new family of chloroquinolinate lanthanoid complexes of the formula A^+^[Ln(5,7Cl_2_q)_4_]^–^, with Ln = Y^3+^, Tb^3+^ and Dy^3+^ and A^+^ = Na^+^, NEt_4_^+^ and K_0.5_(NEt_4_)_0.5_^+^, is studied, both in bulk and as thin films. Several members of the family are found to present single-molecule magnetic behavior in bulk. Interestingly, the sodium salts can be sublimed under high vacuum conditions retaining their molecular structures and magnetic properties. These thermally stable compounds have been deposited on different substrates (Al_2_O_3_, Au and NiFe). The magnetic properties of these molecular films show the appearance of cusps in the zero-field cooled curves when they are deposited on permalloy (NiFe). This indicates a magnetic blocking caused by the interaction between the single-ion magnet and the ferromagnet. X-ray absorption spectroscopy confirms the formation of hybrid states at the molecule/metal interface.

## Abstract 2

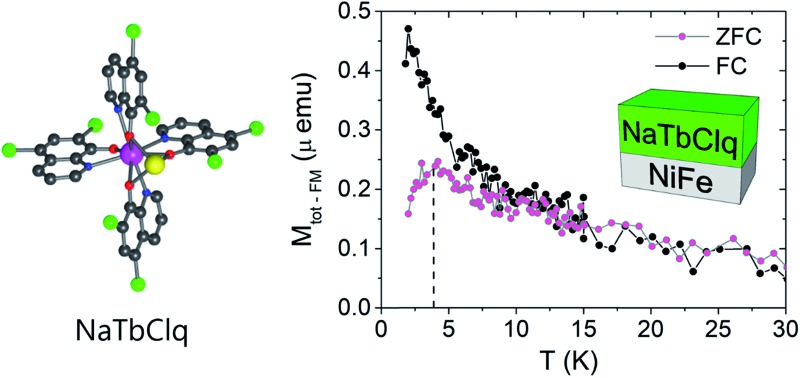
Magnetic analogues of Alq_3_ give rise to molecular/ferromagnetic interfaces with specific hybridization, opening the door to interesting spintronic effects.

## Introduction

The integration of molecules and molecule-based materials in spintronic devices has opened a new field, known as molecular spintronics, which aims at developing a new generation of molecular devices. To reach this goal, the unique possibilities offered by molecular systems to perform electronic functions, to form self-organized nanostructures and to exhibit quantum effects at the nanoscale are exploited.^1^–^3^ These devices are typically formed by the insertion of a thin layer of molecules, or a single-molecule at the limit of miniaturization, in between two metallic electrodes, which are often ferromagnetic in order to inject spin polarized charges into a molecular system acting as a spin collector or as a spin filter. The fabrication of such devices largely depends on the capability of the molecules to remain intact when deposited on the surface electrode.

Owing to its electronic versatility, coordination chemistry is the major source of molecules in this context.^4^ Still, in contrast to what happens with organic molecules, these molecular complexes are often chemically unstable in the presence of a metallic substrate (since they often undergo redox reactions). Furthermore, they are not always compatible with the high-vacuum technologies typically used in spintronics, since in most cases they are forming salts that cannot be sublimed. Hence, a current focus of interest in this area is the design of robust molecular coordination complexes that are both chemically stable when they are in direct contact with the device components and that survive the high-vacuum processing techniques required to fabricate the device. In many cases, this requires the design of thermally stable molecular complexes and, in some other cases, the functionalization of the molecule in order to tune its interactions with the device components and its chemical stability. Notice however that, although here we focus on a sublimation approach, a solution-based approach has also been shown to provide remarkable examples in molecular spintronics, using, in particular, carbon materials (graphene and carbon nanotubes) as substrates.^3^,^5^,^6^

The number of thermally stable molecular complexes that have been experimentally probed to be suitable for spintronics remains very low. A relevant example is provided by Alq_3_ (tris-(8-hydroxyquinolinato)aluminium), which is the most used molecule in the construction of molecular spin valves.^7^,^8^ This quinolinato-based complex was introduced in molecular spintronics after having had a high impact in molecular electronics, where it was widely studied in organic light emitting diodes,^9^–^11^ organic solar cells^12^,^13^ and organic field effect transistors.^14^ In view of their robustness and flat geometry, phthalocyanine molecules have also been extensively deposited on a variety of substrates,^15^,^16^ including ferromagnetic cobalt. Some of these molecules are magnetic and therefore their magnetic moment can be polarized in the presence of a ferromagnetic surface. This is the case for a CoPc molecule deposited on cobalt, which exhibits spin polarization effects as a result of the strong hybridization with the cobalt surface.^17^

Another important class of magnetic molecules that has also emerged in this context are the so-called single-molecule magnets (SMMs) and, in particular, mononuclear complexes based on lanthanoids.^18^ The archetypical example is provided by the family of bis-phtalocyaninato lanthanoid complexes with the formula LnPc_2_. Due to its chemical robustness and thermal stability, this family of complexes has been extensively deposited on surfaces by sublimation. Thus, Malavolti *et al.*^19^ deposited TbPc_2_ complexes onto ferromagnetic substrates (LSMO and cobalt) and Klar *et al.*^20^ deposited DyPc_2_ and TbPc_2_ complexes onto highly ordered pyrolytic graphite. Other lanthanoid-based SMMs have been reported by Dreiser *et al.*,^21^ who deposited the mononuclear Er(trensal) complex (where trensal = 2,2′,2′′-tris-(salicylideneimino)triethylamine) onto graphene; Kiefl *et al.*,^22^ who studied a polynuclear SMM system [Dy(hfac)_3_(PyNO)]_2_ deposited on gold, (where hfac = hexafluoroacetylacetonate and PyNO = pyridine-*N*-oxide); and Gao *et al.*,^23^,^24^ who studied the magnetic properties and thermal stability of some lanthanide complexes, like Dy(fod)_3_(bpy) (where fod = 1,1,1,2,2,3,3-heptafluoro-7,7-dimethyl-4,6-octadione and bpy = 2,2-bipyridine) and [ADyL_4_]·[solvent] (where L = 4-hydroxy-8-methyl-1,5-naphthyridine-3-carbonitrile and A an alkali metal ion (A = Na, K, Rb, Cs)). Other SMMs based on polynuclear transition metal complexes such as Fe_4_(Ph–C(CH_2_O)_3_)_2_(dpm)_6_, where dpm = dipivaloylmethane, (in short Fe_4_)^25^ and {[(CH_3_)_2_CHCH_2_]_2_NH_2_}{Cr_7_NiF_8_[O_2_CC(CH_3_)_3_]_16_} (in short Cr_7_Ni)^26^ have also been sublimed and deposited, but only on gold substrates. In general, most of these systems have been shown to maintain their molecular integrities and magnetic properties when sublimed on surfaces. Still, some differences with the bulk have been observed in the spin dynamics of Dy(fod)_3_(bpy) and Fe_4_ films, which have shown significant changes in their anisotropy barriers. In some cases, the interactions of the surface with the molecules can affect the intrinsic properties of the molecule, which are found to be modified due to the interaction between the orbitals of the substrate and the molecule *via* redox reactions or magnetic exchange interactions, as suggested by several authors.^15^,^27^–^30^

From these results, one can conclude that the deposition under vacuum of thermally stable SMMs on surfaces is in its infancy. Only in a few cases have these molecules been deposited on ferromagnetic (FM) substrates, and even less effort has been devoted to integrate these SMMs in spintronic devices. Our group has initiated this kind of research using lanthanoid-based quinolinato complexes as SMMs. The first report in this context was the fabrication of spin valves containing trinuclear complexes as the spin collector with the formula Ln_3_q_9_ (where Ln = Y^3+^ and Tb^3+^ and *q* = 8-hydroxyquinolinato),^27^ whereas the magnetic properties of Dy and Tb complexes were previously reported by Chilton *et al.*^31^ These neutral molecules can be sublimed under high vacuum conditions at temperatures between 250 and 350 °C and a base pressure of 5 × 10^–11^ mbar. The resulting spin valves showed spin-polarized electron transport across molecular states, supported by the coexistence of thermally activated transport and a robust magnetoresistance effect at room temperature.^32^ However, these trinuclear complexes have shown some reactivity when deposited directly on metallic surfaces. For example, they readily react with a copper surface to form interfacial mixed metal oxides.^33^

In the present work, we report the preparation and characterization of thermally stable lanthanoid-based mononuclear complexes, analogues to Alq_3_, with the final aim of integrating them in spintronic devices. In molecular magnetism, the interest for this kind of magnetic molecule is twofold. On the one hand, they are expected to be more stable and easier to sublime than the trinuclear lanthanoid species. On the other hand, they are much simpler from the electronic and magnetic points of view since they only contain a single site to accommodate the lanthanoid ion. In the first section we show that the use of a quinolinato derivative, the 5,7-dichloro-8-hydroxyquinolinato monoanion (in short 5,7Cl_2_q), yields a family of mononuclear lanthanoid complexes of the general formula A^+^[Ln(5,7Cl_2_q)_4_]^–^, with Ln = Y^3+^, Tb^3+^ and Dy^3+^ and A^+^ = Na^+^, NEt_4_^+^ and K_0.5_(NEt_4_)_0.5_^+^, which behave as SMMs. In the second section we test the capability of these magnetic molecules to be deposited under UHV conditions on various substrates, including magnetic electrodes, while keeping their molecular structures intact and maintaining their magnetic behaviour.

## Experimental

All materials and reagents were purchased from Sigma-Aldrich and used as received, except for the ligand 5,7-dichloro-8-hydroxyquinoline (5,7Cl_2_q) which was recrystallized in chloroform.

### Synthesis of Na[Ln(5,7Cl_2_q)_4_] (Ln = Y^III^ (**1**), Tb^III^ (**2**), and Dy^III^ (**3**))

856 mg (4 mmol) of the ligand 5,7Cl_2_q was dissolved in 80 ml of absolute ethanol at 50 °C. A solution of 160 mg of NaOH (4 mmol) dissolved in 40 ml of absolute ethanol at 50 °C was added. Then, a solution of hot absolute ethanol (50 °C) containing LnCl_3_.6H_2_O (0.6 mmol) was added dropwise and kept under stirring at 50 °C for 1 hour and then cooled to room temperature. The yellow precipitate was filtered on a sintered-glass filter and washed with milli-pore water and 10 ml of a cold mixture of 1 : 1 EtOH : H_2_O. The phase purity was established by X-ray powder diffraction. Anal. calc. NaYClq: C_36_H_16_O_4_N_4_Cl_8_YNa (964.06): C, 44.9; H, 1.7; N, 5.8%. Found: C, 43.8; H, 1.1; N, 5.6%; EDX: Na/Y = 1, Cl/Y = 8, found Na/Y = 0.85, Cl/Y = 8.25. Anal. calc. NaTbClq: C_36_H_16_O_4_N_4_Cl_8_TbNa (1034.08): C, 41.8; H, 1.6; N, 5.4%. Found: C, 41.7; H, 0.9; N, 5.2%; EDX: Na/Tb = 1, Cl/Tb = 8, found Na/Tb = 0.89, Cl/Tb = 7.71. Anal. calc. NaDyClq: C_36_H_16_O_4_N_4_Cl_8_DyNa (1037.65): C, 41.7; H, 1.6; N, 5.4%. Found: C, 41.6; H, 1.0; N, 5.2%; EDX: Na/Dy = 1, Cl/Dy = 8, found Na/Dy = 0.91, Cl/Dy = 7.36. FTIR *ν* (cm^–1^): 958 (m) C–Cl (**1**), 957 (m) C–Cl (**2**) and 957 (m) C–Cl (**3**). ES-MS in negative mode; *m*/*z* = 941 [Y(5,7-Br_2_q)_4_]^–^ anion for **1**, *m*/*z* = 1011 [Tb(5,7-Br_2_q)_4_]^–^ anion for **2** and *m*/*z* = 1012 [Dy(5,7-Br_2_q)_4_]^–^ anion for **3**.

### Synthesis of NEt_4_[Dy(5,7Cl_2_q)_4_] (**4**)

856 mg (4 mmol) of the ligand 5,7Cl_2_q and 662 mg (4 mmol) of NEt_4_Cl were dissolved in 80 ml of absolute ethanol at 50 °C under stirring. 556 ml (4 mmol) of Et_3_N was added. Then, a solution of hot absolute ethanol (50 °C) containing DyCl_3_·6H_2_O (0.6 mmol) was added dropwise and kept under stirring at 50 °C for 1 hour and then cooled to room temperature. The yellow precipitate was filtered on a sintered-glass filter and washed with milli-pore water and 10 ml of a cold mixture of 1 : 1 EtOH : H_2_O. The phase purity was established by X-ray powder diffraction. Anal. calc. NEtDyClq: C_44_H_36_O_4_N_5_Cl_8_Dy (1144.91): C, 46.2; H, 3.2; N, 6.1%. Found: C, 46.9; H, 2.6; N, 6.0%; EDX: Cl/Dy = 8, found Cl/Dy = 7.23. FTIR *ν* (cm^–1^): 957 (m) C–Cl. ES-MS in negative mode; *m*/*z* = 1012 [Dy(5,7-Br_2_q)_4_]^–^ anion for **4**.

### Synthesis of K_0.5_(NEt_4_)_0.5_[Dy(5,7Cl_2_q)_4_] (**5**)

856 mg (4 mmol) of the ligand 5,7Cl_2_q, 331 mg (2 mmol) of NEt_4_Cl and 200 mg (2 mmol) of KNO_3_ were dissolved in 80 ml of absolute ethanol at 50 °C under stirring. 556 ml (4 mmol) of Et_3_N was added. Then, a solution of hot absolute ethanol (50 °C) containing DyCl_3_.6H_2_O (0.6 mmol) was added dropwise and kept under stirring at 50 °C for 1 hour and then cooled to room temperature. The yellow precipitate was filtered on a sintered-glass filter and then washed with milli pore water and 10 ml of a cold mixture of 1 : 1 EtOH : H_2_O. The phase purity was established by X-ray powder diffraction. Anal. calc. KNEtDyClq: C_80_H_52_O_8_N_9_Cl_16_Dy_2_K (2198.67): C, 43.7; H, 2.4; N, 5.7%. Found: C, 41.8; H, 1.4; N, 5.6%; EDX: K/Dy = 0.5, Cl/Dy = 16, found Na/Dy = 0.38, Cl/Dy = 17.28. FTIR *ν* (cm^–1^): 954 (m) and 955 (m) C–Cl. ES-MS in negative mode; *m*/*z* = 1012 [Dy(5,7-Br_2_q)_4_]^–^ anion for **5**.

### Single crystal diffraction

Single crystals of **3**, **4**, and **5** were obtained after recrystallization in DMF for **3**, and for **4** and **5** suitable crystals were obtained after recrystallization in acetonitrile. Compound **4** is isostructural to the previously reported Nd analogue.^34^ Each single crystal was mounted on a cryoloop using a viscous hydrocarbon oil to coat the crystal. X-ray diffraction data were collected at 120 K on a Supernova diffractometer equipped with a graphite-monochromated Enhance (Mo) X-ray Source (*λ* = 0.71073 Å). The program CrysAlisPro, Oxford Diffraction Ltd., was used for the unit cell determinations and data reduction. Empirical absorption correction was performed using spherical harmonics, implemented using the SCALE3 ABSPACK scaling algorithm. The crystal structures were solved and refined against all of the *F*^2^ values by using the SHELXTL suite of programs. Non-hydrogen atoms were refined anisotropically and hydrogen atoms were placed at calculated positions (riding model). A summary of the data collection and structure refinements is provided in Table SI1, ESI.† A structure was obtained from the X-ray diffraction data obtained for the crystal of **5**, with a poor *R*-factor (21.56%); however, the composition of the structure was corroborated using elemental analysis, EDX and mass spectrometry. The X-ray crystallographic coordinates for the structures reported in this article have been deposited at the Cambridge Crystallographic Data Centre (CCDC), under the deposition numbers CCDC-; 1557647 (**3**), CCDC-; 1557649 (**4**), and CCDC-; 1557648 (**5**).†

### Physical measurements

#### CHN-EA

For the CHN elemental analysis an EA 1110 CHNS-O elemental analyzer from CE instruments was utilized. EDX: Energy-dispersive X-ray spectroscopy was carried out with a FEI/Philips XL-30 Field Emission ESEM for the five powdered compounds. ESI-MS: Electrospray ionization mass spectrometry was carried out with a Waters Micromass ZQ spectrometer. Isotopic patterns were analyzed using the Mass software. XRPD: X-ray powder diffraction was carried out with a PANalytical Empyrean X-ray powder diffractometer with Cu radiation from Oxford Cryostream with the PIXcel detector XRPD for the capillary measurement. IR: Infrared spectra of the powdered compounds and layers were recorded in transmission mode on a Nicolet 5700 FT-IR spectrometer. TGA: Thermogravimetric analysis was performed using a thermal analyzer model Mettler Toledo TGA/SDTA 851e that operates in the range [25, 1100] °C and has a sensitivity of 0.1 μg.

### SIMPRE software

For the theoretical calculations, we have used the SIMPRE computational package,^35^,^36^ introducing the atomic coordinates and the magnetic properties of the compounds as an input and modifying the two fitting parameters (*D*_r_ and *Z*_i_) of the REC model. A detailed explanation is provided in the ESI.†

### Magnetic measurements of the bulk compounds

Magnetic measurements were performed on the powdered compounds in a Quantum Design Physical Property Measurement System (PPMS). The AC susceptibility magnetic measurements were performed between 10 and 40 K at different frequencies under an oscillating field of 3.95 Oe. For comparison with the sublimed material, the magnetic characterization of the bulk NaDyClq was measured in a state-of-the-art SQUID magnetometer (Quantum Design MPMS), with a sensitivity of 5 × 10^–8^ emu, in which several NaDyClq films on glass were scratched and measured as a powder.

### Compound sublimation and characterization

The Na[Ln(5,7Cl_2_q)_4_] and the Na[Ln(5,7Cl_2_q)_4_] (Ln = Tb, Dy)/metal hybrid layers were fabricated *in situ* in a dual-chamber evaporator (*p*_base_ = 5 × 10^–11^). The metallic layers (Au, NiFe, Co) were deposited *via* e-beam evaporation, while the molecular layers were sublimed thermally from conventional effusion cells, at 340 °C, keeping the substrate at a constant temperature of 25 °C. For comparison between the bulk and sublimed NaDyClq, the compound was evaporated on a glass substrate and then scratched. In the bilayer cases, the metallic layer thickness was set to 8–10 nm, and the NaLnClq molecular layer thickness was varied between 10 and 15 nm. EDX: Energy-dispersive X-ray spectroscopy was carried out with a FEI/Philips XL-30 Field Emission ESEM for the five different films. IR: Infrared spectra of the powdered compounds and layers were recorded in transmission mode for the five different films deposited on a glass substrate using a Nicolet 5700 FT-IR spectrometer. MALDI-TOF: The molecular compounds were sublimed on SiO_2_–Si substrates. The resulting films of Na[Ln(5,7Cl_2_q)_4_] were analysed in a 5800 MALDI TOF/TOF (ABSciex) in negative (and positive) reflection mode (3000 shots for every position) in a mass range of 800–5500 *m*/*z*. No matrix was used. AFM: Surface topographies were imaged using an Atomic Force Microscope (Agilent 5500), operating in tapping mode with a tip frequency of 342 kHz. Cantilevers were made of silicon, with a spring constant of 40 Nm^–1^. The image area amounts to 1 μm × 1 μm.

### Magnetic measurements of the films

The magnetic properties of the hybrid layers were characterized in a state-of-the-art SQUID magnetometer (Quantum Design MPMS), with a sensitivity of 5 × 10^–8^ emu. The ferromagnetic contribution in the molecule/ferromagnet bilayers was subtracted by using a reference sample.

### X-ray absorption spectroscopy

The XAS measurements were carried out at the beamlines I1011 in the Max Lab Synchrotron (Lund) and BOREAS in the ALBA Synchrotron (Barcelona) equipped with *in situ* preparation chambers (10^–10^ mbar) where the NaDyClq/metal layers were prepared. The molecules were grown from effusion cells and the NiFe was e-beam evaporated. The X-ray absorption measurements were taken in total electron yield, with the spectra normalized to a freshly sputtered Au-grid.

## Results and discussion

### Chemical design and structural characterization of the magnetic molecules

**Fig. 1:**
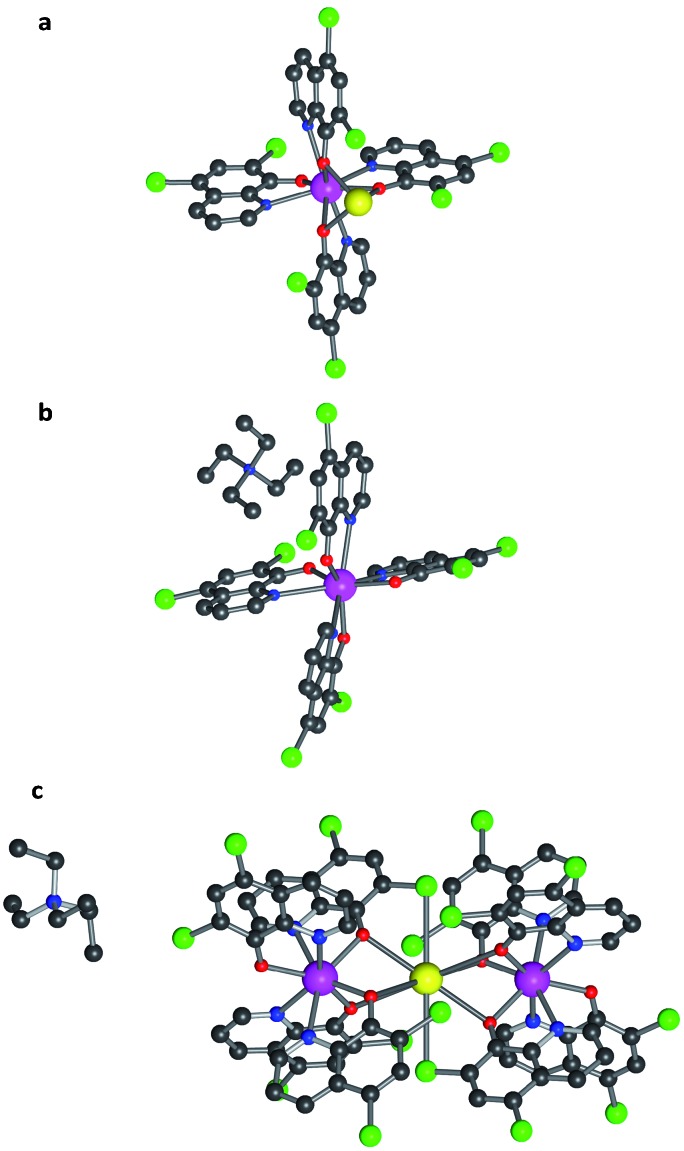
The molecular structure of the compounds. (a) Na[Ln(5,7Cl_2_q)_4_] (in short NaLnClq with Ln = Y (**1**), Tb (**2**) or Dy (**3**)); (b) NEt_4_[Dy(5,7Cl_2_q)_4_] (**4**); (c) K_0.5_(NEt_4_)_0.5_[Dy(5,7Cl_2_q)_4_] (**5**). The central lanthanoid ion is in pink, oxygen in red, nitrogen in blue, carbon in black, chlorine in green and sodium (in a) and potassium (in c) in yellow. Hydrogen atoms have been omitted for clarity.

In contrast to what happens for Al^3+^, when a lanthanide ion reacts with 8-hydroxyquinoline the trinuclear species Ln_3_q_9_ is formed as a major product. A possible way to favour the formation of mononuclear lanthanoid complexes is to introduce bulky substituents into the 8-hydroxyquinolinato ligand with the aim of increasing its steric hindrance. Following this approach, we have used a quinoline derivative, the 5,7-dichloro-8-hydroxyquinolinato monoanion (in short 5,7Cl_2_q). The result was the isolation of mononuclear anionic species [Lnq_4_]^–^. Thus, using A^+^ = Na^+^, NEt_4_^+^ and K_0.5_(NEt_4_)_0.5_^+^ as the counterions, we have prepared several salts containing [Lnq_4_]^–^. Specifically, we have isolated crystalline materials of the series Na[Ln(5,7Cl_2_q)_4_] with Ln^3+^ = Y (**1**), Tb (**2**) and Dy (**3**), as well as the dysprosium derivative of the series NEt_4_[Ln(5,7Cl_2_q)_4_] (**4**) and K_0.5_(NEt_4_)_0.5_[Ln(5,7Cl_2_q)_4_] (**5**). After recrystallization in DMF of **1**, **2** and **3**, and in MeCN for **4** and **5**, single crystals suitable for X-ray diffraction were obtained for the three series (Table SI1, ESI†). In all of the compounds, each lanthanoid is coordinated to four 5,7Cl_2_q ligands, each of them acting as a bidentate ligand *via* the nitrogen and oxygen atoms (Fig. 1). The coordination environment of each LnO_4_N_4_ centre can be described as bicapped trigonal prismatic (**3** and **5**) and distorted square antiplanar (**4**) (see Fig. 1 and SI2, ESI†). In these compounds, the alkali metal ions (Na^+^ and K^+^ in **3** and **5**, respectively) are placed nearby the lanthanoid ion (*d*_Dy–Na_ = 3.323(4) Å and *d*_Dy–K_ = 3.98(2) Å), while the bulky NEt_4_^+^ in **4** is simply acting as a counterion, being far from the lanthanoid. Thus, in the Na^+^ series, the cation is bonded to three oxygen atoms from three 5,7Cl_2_q ligands, and in the K_0.5_(NEt_4_)_0.5_ series the K^+^ is trapped by two [Lnq_4_]^–^ complexes forming Ln–K–Ln trimers, wherein K^+^ is coordinated to six oxygen atoms and two axial chlorine atoms from six different quinolinato ligands. The ESI-MS measurements showed the existence of the [Ln(5,7Cl_2_q)_4_]^–^ anion in all of the compounds, confirming their stability.

### Magnetic properties

**Fig. 2:**
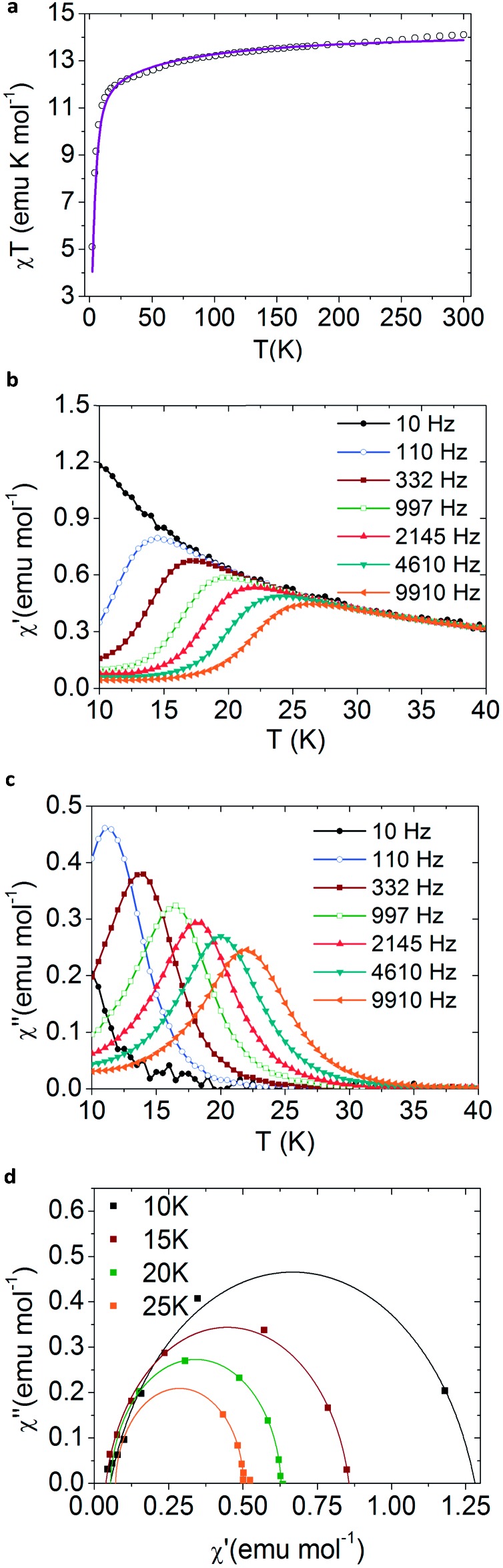
Magnetic measurements for bulk NaDyClq (**3**). (a) *χT vs. T* plot at 1000 Oe, where the solid line is the theoretical simulation obtained using SIMPRE software. (b) The frequency dependence of the in-phase magnetic susceptibility under a 500 Oe DC field. (c) The frequency dependence of the out of phase magnetic susceptibility under a 500 Oe DC field. (d) Cole–Cole plots where the solid lines are fits to eqn SI7, ESI.†

Fig. 2a and SI5, ESI† show the static DC magnetic measurements for the magnetic compounds **2**, **3**, **4** and **5**, plotted as *χT vs. T*. The *χT* values at 300 K in the four complexes are near those expected for the ^7^F_6_ and ^6^H_15/2_ multiplets of Tb^III^ (11.81 emu K mol^–1^) and Dy^III^ (14.17 emu K mol^–1^). The characteristic decay observed at low temperatures is caused by the depopulation of the excited Stark levels, which is typical for lanthanoid centres.

A complete description of the ground multiplet crystal field splitting of each compound has been inferred using the Radial Effective Charge (REC) model^37^ in the SIMPRE^35^,^36^ software package (see ESI†). For the Dy derivatives **3**, **4** and **5** this kind of calculation predicts a ground spin doublet *M*_J_ = ±15/2, which is well separated in energy from the rest of the energy levels by more than 49 cm^–1^ in **4**, and more than 100 cm^–1^ in **3** and **5**. For the Tb derivative (**2**) the predicted ground state is *M*_J_ = ±6, which is separated from the first excited level by 165 cm^–1^. These energy schemes for the *M*_J_ levels are in principle compatible with the SMM behaviour observed in both compounds.

The dynamic AC magnetic measurements show frequency-dependent maxima in both *χ*′ and *χ*′′ when applying an external DC field of 500 Oe (Fig. 2b and c, and SI13 and 14, ESI†). Notice that these maxima disappear at zero field possibly due to the presence of a fast relaxation of the magnetization through a quantum tunnelling mechanism, which is duly removed when an external DC field is applied. Under these conditions, **3** shows the highest blocking temperature with *χ*′′ maxima observed up to *T* = 24 K at high frequencies (9910 Hz) (Fig. 2c), while in the other two Dy compounds **4** and **5** these maxima are observed below 4.5 K and 13 K, respectively (see Fig. SI14, ESI†). In the Tb derivative (**2**) a continuous divergence is observed in *χ*′′ at low temperatures, with no maximum detected above 2 K (see Fig. SI13, ESI†). This is a commonly observed feature for Tb^III^ complexes due to the non-Kramer’s nature of the metal ion.

Fits of the Cole–Cole plots^38^,^39^ for **3** lead to low α-values, in agreement with the existence of a single dominant relaxation mechanism (see Fig. SI7c, 8c, 9c and 10c, ESI†). α is defined as the Cole–Cole parameter and corresponds to the value of the intrinsic relaxation time of the compound. These data were tested by applying either a Raman or an Orbach mechanism hypothesis (eqn SI6 and 7, ESI†). We find that the fit is better for the Raman mechanism (see Table SI8, ESI†). This result is also supported by the fact that the effective energy barrier (calculated from the Arrhenius fit; see Fig. SI11, ESI†) is much lower than the estimated gap between the ground spin doublet and the first excited state (60.1 cm^–1^ compared to 126 cm^–1^).^40^ Thus, all available experimental data can be rationalized by assuming a simple dominant Raman-type relaxation mechanism that masks the Orbach process. In turn, this means that the relaxation is mainly taking place through an exchange of energy by lattice vibrations *via* a virtual level.

### Thermal stability

**Fig. 3:**
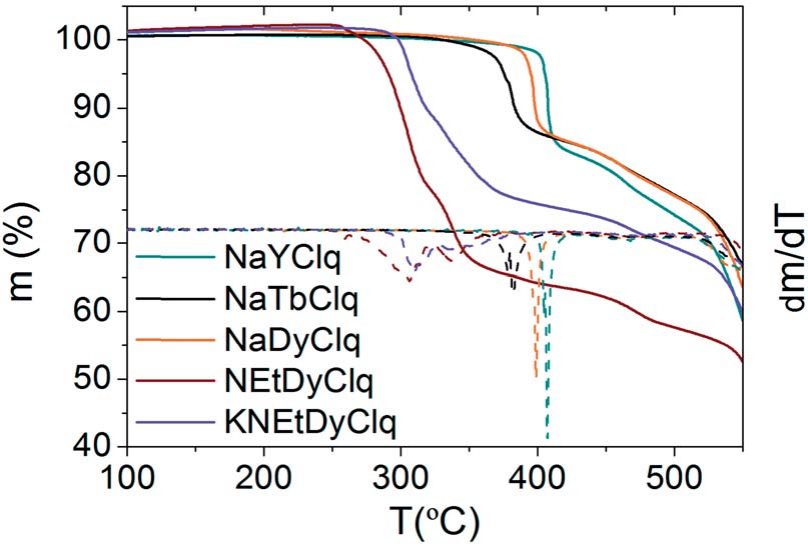
Thermogravimetric analysis (TGA) of the compounds. The dashed lines magnify the changes of slope in *m*(*T*) since they are the derivatives d*m*/d*T*.

Thermogravimetric analysis (TGA) shows that the sodium compounds, **1**, **2** and **3**, present higher thermal stability than the alkyl ammonium derivatives **4** and **5**, with a quasi-vertical change of slope above 400 °C for the sodium derivatives (Fig. 3).

In fact, these sodium derivatives can be sublimed. To check if they retain their molecular structure after sublimation, they were thermally evaporated (*P*_base_ = 10^–11^ mbar and *T*_subl_ = 260–340 °C). The result was a deposition of yellowish layers, which were subsequently investigated by EDX, IR and MALDI-TOF techniques (see Methods and Fig. SI16, ESI†). This multi-technique process indicated that the sodium derivatives **1**, **2** and **3** maintain their molecular integrity upon sublimation. However, the absence of the correct pattern in the MALDI-TOF spectra for the films of **4** and **5** revealed that they were not sublimable, since the molecular structure of these molecules is lost. Such differences can be rationalized by close inspection of the crystal structures. In the sodium derivatives, the cation is tightly bound to the [Ln(5,7Cl_2_q)_4_]^–^ anion (*via* tridentate oxygen coordination), thus yielding a neutral molecule that can easily sublime as a whole entity. On the contrary, in **4** the organic cation, NEt_4_^+^, is only very weakly bound to the [Ln(Cl_2_q)_4_]^–^ anion, meaning they cannot sublime together, whereas in **5** the K^+^ cation is tightly bound to two [Ln(Cl_2_q)_4_]^–^ moieties giving rise to a trinuclear anion, which is again only very weakly bound to its neighbouring NEt_4_^+^ cation (see Fig. 1).

**Fig. 4:**
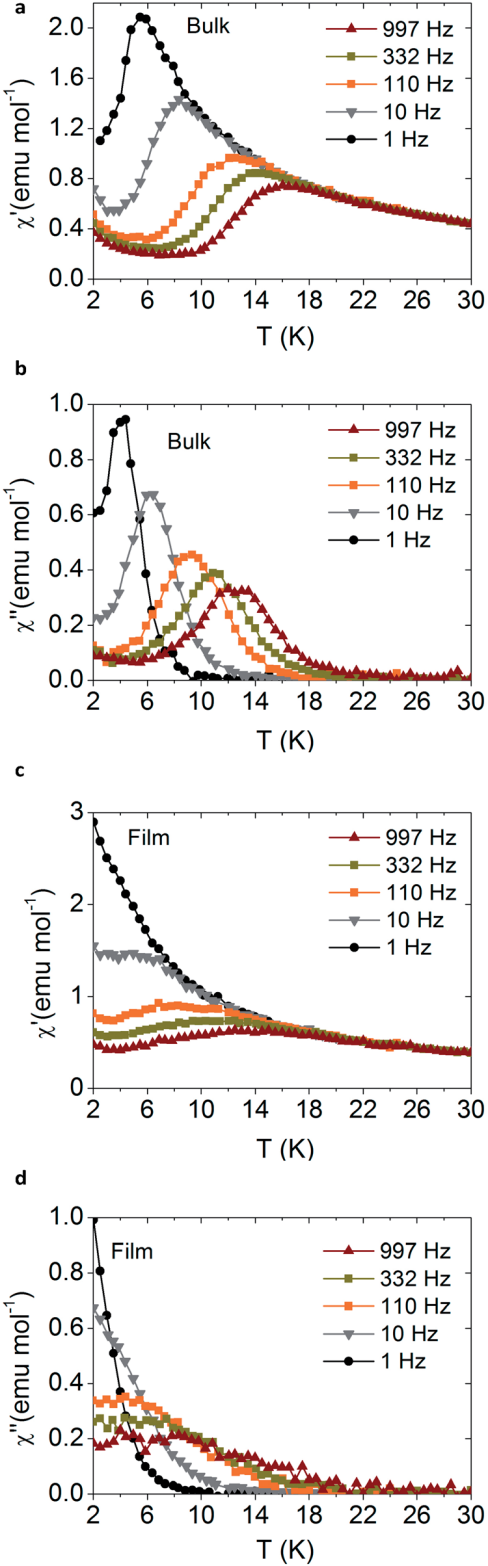
AC magnetic measurements of **3** (NaDyClq), in bulk (a and b) and as a film (c and d) showing the preservation of the slow relaxation of the magnetization. The measurements were performed under a 1000 Oe external DC field.

Magnetic measurements also support the fact that the molecular structure of the sodium derivatives is preserved upon sublimation. Fig. 4 shows the magnetic AC magnetic measurements for the NaDyClq compound, in bulk and as a film, measured by SQUID magnetometry. We note that the SMM behaviour, characterized by a slow relaxation of the magnetization at low temperatures, is preserved in the film. The small differences observed between the bulk and the film may be due to the fact that while the starting material is polycrystalline, it becomes amorphous after sublimation; as a consequence, a small change in the blocking temperature is expected, as mentioned previously by Dreiser *et al.*^41^

We should point out the fact that, although rare, other thermally stable sublimable ionic molecules based on mononuclear lanthanoid complexes are known. Among them, the tetrakis–diketonato complexes of the type M[Ln(dik)_4_] where M = Cs, Y and La^42^–^44^ stand out, since some of these molecules have been used in the fabrication of nano-optical devices by thermal techniques under high vacuum conditions.^45^ These compounds are non-hygroscopic and air-stable and can be sublimed on substrates maintaining their molecular structure.^43^,^44^ Sublimable ionic molecules based on mononuclear complexes with the 8-hydroxyquinolinato ligand are also known. We also mention the series Li[Ln(q)_4_] (Ln = La; Y and La; Y and Lu)^46^ and the compound Na[Er(q)_4_],^47^ which have been thermally deposited on ITO and gold, and used in the fabrication of OLEDs, thus corroborating the robustness of the quinolato complexes.

### Deposition on ferromagnetic substrates

With the aim of studying a possible interaction between the magnetic molecules and a ferromagnetic substrate, we have sublimed the NaYClq (**1**), NaTbClq (**2**) and NaDyClq (**3**) complexes on different magnetic and non-magnetic substrates to form bilayers. As substrates, we have used gold (Au) and permalloy (NiFe), as well as an insulating layer of Al_2_O_3_. The morphology of the films has been studied by means of atomic force microscopy (AFM). Fig. SI15, ESI† shows the images of the bilayers of NiFe (8 nm)/NaYClq (**1**) (10 nm), NiFe (8 nm)/NaDyClq (**3**) (10 nm) and Co (10 nm)/NaDyClq (**3**) (15 nm). The topography images show a good coverage in all cases. Both NaYClq and NaDyClq show a modest peak to peak and low RMS value for the films of 10 nm on NiFe (*ρ*_ptp_ (NaYClq) = 3.79 nm and *ρ*_RMS_ (NaYClq) = 0.39 nm; *ρ*_ptp_ (NaDyClq) = 2.93 nm and *ρ*_RMS_ (NaDyClq) = 0.32 nm). Besides, the surfaces show grain structures and are uniform. For a thicker NaDyClq film, the surface is smoother and less grain structured; in this case, the *ρ*_RMS_ is still very low (*ρ*_RMS_ = 0.58 nm), despite the appearance of aggregations that increase the *ρ*_ptp_ considerably. These very low roughness values – below 1 nm – make these molecules potential materials to be included in multi-layered vertical devices.

**Fig. 5:**
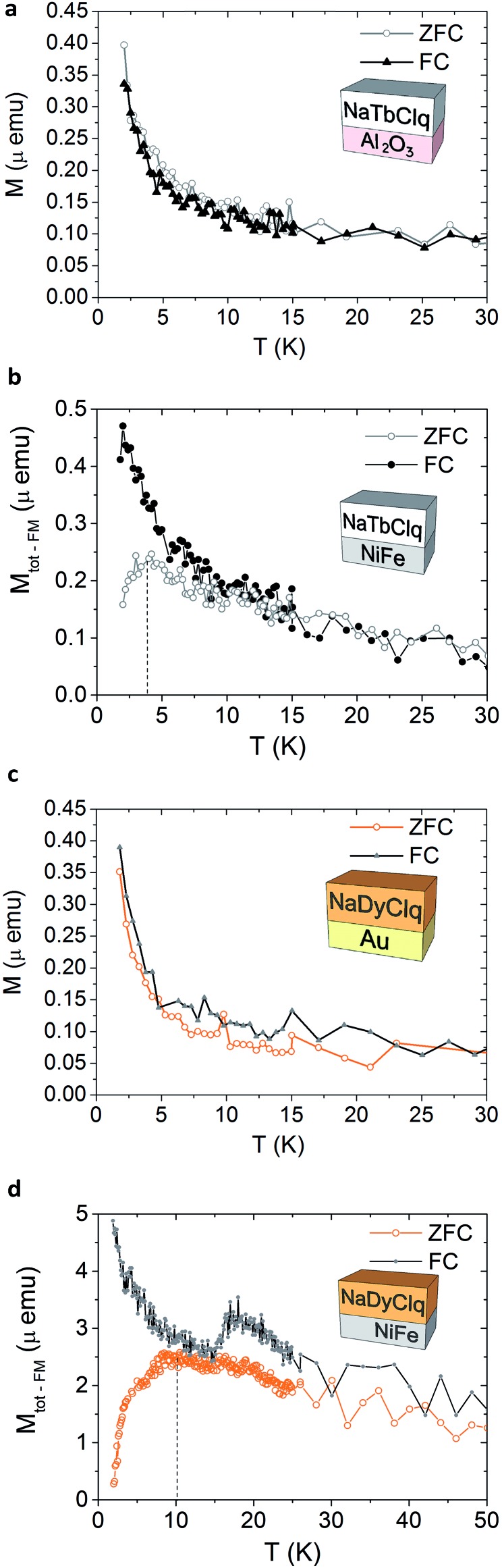
Temperature-dependent magnetization of a 10 nm NaTbClq (**2**) layer grown on a 1.5 nm layer of Al_2_O_3_ (a) and on a 10 nm layer of NiFe (b). Temperature-dependent magnetization of a NaDyClq (**3**) layer grown on a 10 nm layer of Au (c) and on a 10 nm layer of NiFe (d). Note that the displayed magnetization in (a) and (c) is the as-obtained value, while for (b) and (d) the ferromagnetic contribution of the NiFe substrate has been subtracted to visualize the magnetization related to the molecular layer (see Fig. SI17, ESI† for a detailed description).

The magnetic properties of the bilayers have been characterized by measuring the zero-field cooled (ZFC) and field cooled (FC) magnetization (in the presence of a DC field of 500 Oe). Temperature-dependent magnetization of a NaTbClq (**2**) layer grown on Al_2_O_3_ and NiFe (Fig. 5a and b) highlights the influence of the substrate on the magnetic properties of the layers. While the molecules grown on Al_2_O_3_ retain their paramagnetic behaviour, being FC and ZFC curve coincident, for the NaTbClq (**2**)/NiFe bilayer a cusp in the ZFC scan is observed at *ca.* 4 K. Such irreversibility may be associated with magnetic blocking behaviour at this temperature, which is most probably due to the interface coupling between the underlying ferromagnet and the paramagnetic Tb-spins of the molecule. The same holds for NaDyClq (**3**). Thus, on Au this molecular compound shows a paramagnetic behaviour down to 2 K (Fig. 5c), whereas on NiFe it shows a broad cusp in the ZFC scan at *ca.* 10 K, which supports a magnetic blocking (Fig. 5d) around this temperature. In this last case, the blocking temperature is higher, in full agreement with the higher value of the activation energy required for the magnetization reversal in the Dy molecule (as compared with the Tb molecule; see the section on the magnetic properties).

**Fig. 6:**
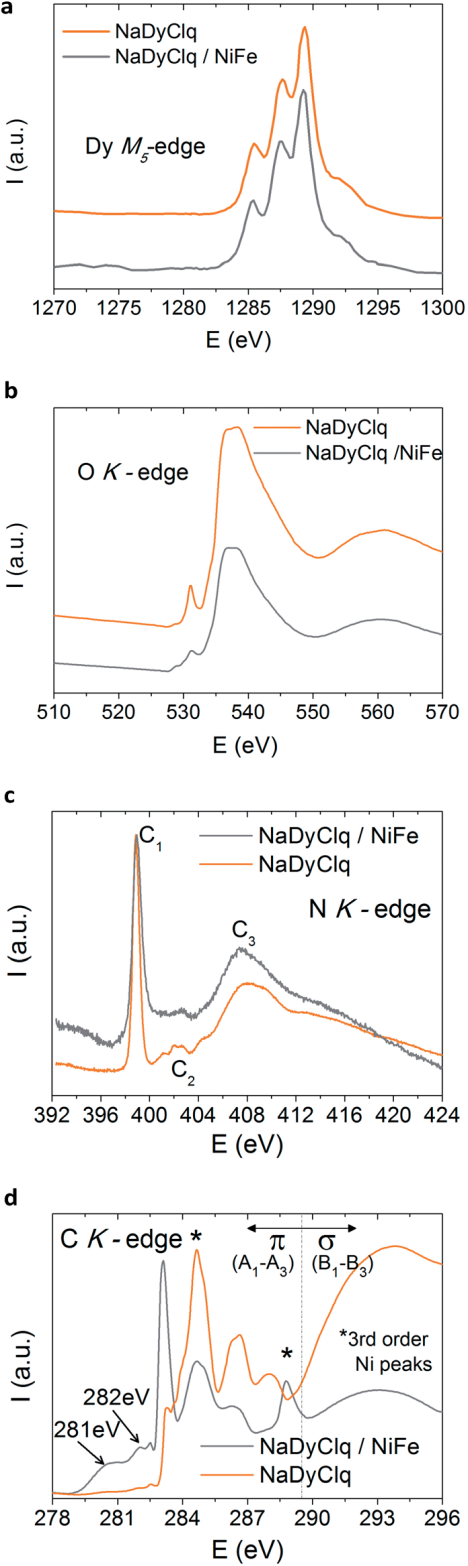
X-ray absorption spectroscopy (XAS) spectrum of the molecule NaDyClq (**3**) compared to that of a NaDyClq/NiFe interface: (a) Dy M_5_-edge, (b) O K-edge, (c) N K-edge and (d) C K-edge.

We can tentatively discuss the nature of the interaction between these molecules and the ferromagnetic surface. A possibility would be simply that the stray magnetic field in the vicinity of the ferromagnetic surface causes a suppression of the quantum tunneling in the SIM, while polarizing its magnetic moment. In addition to this through-space magnetic interaction, one can also consider an electronic interaction between the molecule and the surface leading to the formation of specific hybrid interfacial states with possible charge transfer, resulting in spin polarization of the molecule. This last possibility has been examined by *in situ* X-ray absorption spectroscopy (XAS) on a **3**/NiFe interface. One observes that the XAS spectra of the Dy and O edges are not affected by the ferromagnetic surface (Fig. 6a and b). The same happens for the N K-edge of the pristine molecule that shows three characteristic peaks corresponding to the transitions π (C1) and σ (C2 and C3),^42^ which are maintained in the bilayer. In contrast, the C K-edge of the pristine molecule (in orange in Fig. 6d) is strongly affected by the presence of the FM surface. Thus, the pristine molecule shows three characteristic peaks that are ascribed to the LUMO and LUMO^+1^ transitions localized at the carbon rings of the ligands.^48^,^49^ The two peaks highlighted with a star in Fig. 6d correspond to a high order replica of the Ni signal and do not belong to the molecule/metal interaction. The interaction of the molecule with NiFe leads to a modification in the C K-edge, where two new peaks located at 281 eV and 282 eV are observed in the pre-edge region. These new peaks, which lie in the HOMO–LUMO gap, contribute to the electronic transport and can be ascribed to the hybrid states at the NaDyClq/NiFe interface.^48^

## Conclusions

In this paper we have shown that sublimable magnetic molecules based on mononuclear lanthanoid complexes, which are magnetic analogues of the well-known Alq_3_ molecule, can be designed and deposited intact on ferromagnetic substrates to form high-quality thin films. This allowed us to prepare hybrid molecular/ferromagnetic interfaces, in which the magnetic molecules maintain their single-molecule magnetic behaviour. Interestingly, when deposited on a ferromagnetic NiFe substrate these molecules undergo magnetic blocking at low temperatures as a consequence of the interface coupling between the underlying ferromagnet and the paramagnetic Ln-spins of the molecule originating at the molecule/metal interface. These features have been shown to be important in the design of spintronic devices based on these molecules. In particular, a sign-inversion in the magneto-resistance of these devices has been observed, which has been attributed to the specific hybridization established at the NaDyClq/NiFe interface.^49^

## Conflicts of interest

There are no conflicts to declare.

## Supplementary Material

Supplementary information

Crystal structure data

## References

[cit1] Camarero J., Coronado E. (2009). J. Mater. Chem..

[cit2] Sanvito S. (2011). Chem. Soc. Rev..

[cit3] Urdampilleta M., Klyatskaya S., Cleuziou J.-P., Ruben M., Wernsdorfer W. (2011). Nat. Mater..

[cit4] Coronado E., Yamashita M. (2016). Dalton Trans..

[cit5] Cervetti C., Heintze E., Bogani L. (2014). Dalton Trans..

[cit6] Cervetti C., Rettori A., Pini M. G., Cornia A., Repollés A., Luis F., Dressel M., Rauschenbach S., Kern K., Burghard M., Bogani L. (2015). Nat. Mater..

[cit7] Wang F., Vardeny Z. V. (2009). J. Mater. Chem..

[cit8] Dediu V., Hueso L. E., Bergenti I., Riminucci A., Borgatti F., Graziosi P., Newby C., Casoli F., De Jong M. P., Taliani C., Zhan Y. (2008). Phys. Rev. B: Condens. Matter Mater. Phys..

[cit9] Garbuzov D. Z., Bulović V., Burrows P. E., Forrest S. R. (1996). Chem. Phys. Lett..

[cit10] Bulović V., Khalfin V., Gu G., Burrows P., Garbuzov D., Forrest S. (1998). Phys. Rev. B: Condens. Matter Mater. Phys..

[cit11] Halls M. D., Schlegel H. B. (2001). Chem. Mater..

[cit12] Song Q. L., Li F. Y., Yang H., Wu H. R., Wang X. Z., Zhou W., Zhao J. M., Ding X. M., Huang C. H., Hou X. Y. (2005). Chem. Phys. Lett..

[cit13] Peumans P., Yakimov A., Forrest S. R. (2003). J. Appl. Phys..

[cit14] Cicoira F., Santato C. (2007). Adv. Funct. Mater..

[cit15] Lodi Rizzini A., Krull C., Mugarza A., Balashov T., Nistor C., Piquerel R., Klyatskaya S., Ruben M., Sheverdyaeva P. M., Moras P., Carbone C., Stamm C., Miedema P. S., Thakur P. K., Sessi V., Soares M., Yakhou-Harris F., Cezar J. C., Stepanow S., Gambardella P. (2014). Surf. Sci..

[cit16] Bazarnik M., Brede J., Decker R., Wiesendanger R. (2013). ACS Nano.

[cit17] Brede J., Wiesendanger R. (2012). Phys. Rev. B: Condens. Matter Mater. Phys..

[cit18] Guo F.-S., Day B. M., Chen Y.-C., Tong M.-L., Mansikkamäki A., Layfield R. A., Goodwin A. P. C., Ortu F., Reta D., Chilton N. F., Mills D. P. (2017). Angew. Chem., Int. Ed..

[cit19] Malavolti L., Poggini L., Margheriti L., Chiappe D., Graziosi P., Cortigiani B., Lanzilotto V., Buatier de Mongeot F., Ohresser P., Otero E., Choueikani F., Sainctavit P., Bergenti I., Dediu V. a., Mannini M., Sessoli R. (2013). Chem. Commun..

[cit20] Klar D., Candini A., Joly L., Klyatskaya S., Krumme B., Ohresser P., Kappler J.-P., Ruben M., Wende H. (2014). Dalton Trans..

[cit21] Dreiser J., Pacchioni G. E., Donati F., Gragnaniello L., Cavallin A., Pedersen K. S., Bendix J., Delley B., Pivetta M., Rusponi S., Brune H. (2016). ACS Nano.

[cit22] Kiefl E., Mannini M., Bernot K., Yi X., Amato A., Leviant T., Magnani A., Prokscha T., Suter A., Sessoli R., Salman Z. (2016). ACS Nano.

[cit23] Gao C., Yang Q., Wang B.-W., Wang Z.-M., Gao S. (2016). CrystEngComm.

[cit24] Bi Y., Chen C., Zhao Y., Zhang Y., Jiang S.-D., Wang B.-W., Han J., Sun J.-L., Bian Z., Wang Z., Gao S. (2016). Chem. Sci..

[cit25] Margheriti L., Mannini M., Sorace L., Gorini L., Gatteschi D., Caneschi A., Chiappe D., Moroni R., de Mongeot F. B., Cornia A., Piras F. M., Magnani A., Sessoli R. (2009). Small.

[cit26] Ghirri A., Corradini V., Bellini V., Biagi R., Del Pennino U., De Renzi V., Cezar J. C., Muryn C. A., Timco G. A., Winpenny R. E. P., Affronte M. (2011). ACS Nano.

[cit27] Wende H., Bernien M., Luo J., Sorg C., Ponpandian N., Kurde J., Miguel J., Piantek M., Xu X., Eckhold P., Kuch W., Baberschke K., Panchmatia P. M., Sanyal B., Oppeneer P. M., Eriksson O. (2007). Nat. Mater..

[cit28] Bernien M., Miguel J., Weis C., Ali M. E., Kurde J., Krumme B., Panchmatia P. M., Sanyal B., Piantek M., Srivastava P., Baberschke K., Oppeneer P. M., Eriksson O., Kuch W., Wende H. (2009). Phys. Rev. Lett..

[cit29] Coronado E., Forment-Aliaga A., Romero F. M., Corradini V., Biagi R., De Renzi V., Gambardella A., del Pennino U. (2005). Inorg. Chem..

[cit30] Moro F., Biagi R., Corradini V., Evangelisti M., Gambardella A., De Renzi V., del Pennino U., Coronado E., Forment-Aliaga A., Romero F. M. (2012). J. Phys. Chem. C.

[cit31] Chilton N. F., Deacon G. B., Gazukin O., Junk P. C., Kersting B., Langley S. K., Moubaraki B., Murray K. S., Schleife F., Shome M., Turner D. R., Walker J. a. (2014). Inorg. Chem..

[cit32] Bedoya-Pinto A., Prima-García H., Casanova F., Coronado E., Hueso L. E. (2015). Adv. Electron. Mater..

[cit33] Coronado E., et al., 2017, in preparation.

[cit34] O’Riordan A., Van Deun R., Mairiaux E., Moynihan S., Fias P., Nockemann P., Binnemans K., Redmond G. (2008). Thin Solid Films.

[cit35] Baldoví J. J., Cardona-Serra S., Clemente-Juan J. M., Coronado E., Gaita-Ariño A., Palii A. (2013). J. Comput. Chem..

[cit36] Baldoví J. J., Clemente-Juan J. M., Coronado E., Gaita-Ariño A., Palii A. (2014). J. Comput. Chem..

[cit37] Baldoví J. J., Borrás-Almenar J. J., Clemente-Juan J. M., Coronado E., Gaita-Ariño A. (2012). Dalt. Trans..

[cit38] Cole K. S., Cole R. H. (1941). J. Chem. Phys..

[cit39] Guo Y.-N., Xu G.-F., Guo Y., Tang J. (2011). Dalton Trans..

[cit40] Gómez-Coca S., Urtizberea A., Cremades E., Alonso P. J., Camón A., Ruiz E., Luis F. (2014). Nat. Commun..

[cit41] Dreiser J. (2015). J. Phys.: Condens. Matter.

[cit42] Drozdov A. and Kuzmina N., Volatile Compounds of Lanthanides, Elsevier Ltd., 2013.

[cit43] Becht M., Dahmen K.-H., Gramlich V., Marteletti A. (1996). Inorg. Chim. Acta.

[cit44] Kuzmina N., Ryazanov M., Malkerova I., Alikhanyan A., Gleizes A. N. (2001). Eur. J. Inorg. Chem..

[cit45] Teixeira K. C., Moreira G. F., Quirino W. G., Legnani C., Silva R. a., Cremona M., Brito H. F., Achete C. a. (2011). J. Therm. Anal. Calorim..

[cit46] Camargo H., Paolini T. B., Niyama E., Brito H. F., Cremona M. (2013). Thin Solid Films.

[cit47] Bisti F., Anemone G., Donarelli M., Penna S., Reale A., Ottaviano L. (2014). Org. Electron..

[cit48] Demasi A., Piper L. F. J., Zhang Y., Reid I., Wang S., Smith K. E., Downes J. E., Peltekis N., McGuinness C., Matsuura A. (2008). J. Chem. Phys..

[cit49] Bedoya-Pinto A., Miralles S. G., Vélez S., Atxabal A., Gargiani P., Valvidares M., Casanova F., Coronado E., Hueso L. E. (2017). Adv. Funct. Mater..

