# Supplementary material for: Sublimable chloroquinolinate lanthanoid single-ion magnets deposited on ferromagnetic electrodes
Source: Chem Sci. 2017 Oct 17;9(1):199–208. doi: 10.1039/c7sc03463f (PMC5869315; doi:10.1039/c7sc03463f)
Supplement: Supplementary file 1 [file SC-009-C7SC03463F-s001.pdf]

Electronic Supplementary Material (ESI) for Chemical Science.
This journal is © The Royal Society of Chemistry 2017

Supporting Information for:

Sublimable chloroquinolinate lanthanoid single-ion magnets deposited on ferromagnetic electrodes

Sara G. Miralles[a], Amilcar Bedoya-Pinto[b,c], José J. Baldoví[a,d], Walter Cañon-Mancisidor[a,e], Yoann Prado[a], Helena Prima-Garcia[a], Alejandro Gaita-Ariño[a], Guillermo Mínguez Espallargas[a], Luis E. Hueso[b,*] and Eugenio Coronado[a,*]

Table of Contents

1 Synthesis	2
2 IR spectra	17
3 Mass Spectrometry	18
4 Radial Effective Charge (REC) model	20
5 AC Measurements	25
6 Film Characterization	32
7 References	34

1. Synthesis

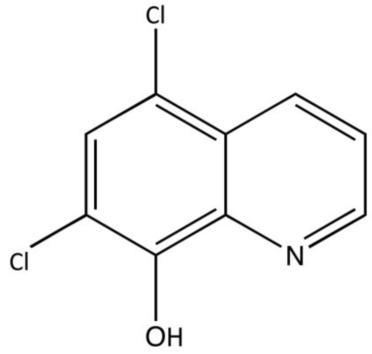

Fig. SI1: *Scheme of the ligand 5,7-dichloro-8-hydroxyquinoline (5,7Cl_2q).*

Table SI1: *Crystallographic data for the compounds.*

Identification code	*NaLnClq* **(3)**	*NEtDyClq* **(4)**	*KNEtDyClq* **(5)**
Empirical formula	$C_{39}H_{23}Cl_8DyN_5NaO_5$	$C_{44}H_{36}Cl_8DyN_5O_4$	$C_{84}H_{58}Cl_{16}Dy_2KN_{11}O_8$
Formula weight	1110.71	1144.88	2280.71
Temperature/K	120(2)	120(2)	120(2)
Crystal system	Monoclinic	Monoclinic	Triclinic
Space group	$P2_1/c$	C2/c	P-1
a/Å	9.9819(2)	36.1061(11)	15.8989(16)
b/Å	19.8256(4)	11.5705(3)	16.0286(16)
c/Å	20.6783(4)	22.7831(6)	19.542(2)
α/°	90	90	98.384(9)
β/°	91.212(2)	109.429(3)	91.201(9)
γ/°	90	90	118.115(10)
Volume/Å^3	4091.26(14)	8976.0(4)	4322.4(8)
Z	4	8	2
ρ_{calc}g/cm^3	1.803	1.694	1.752
μ/mm^{-1}	2.411	2.191	2.322
F(000)	2180	4552	2252
Crystal size/mm^3	0.23 × 0.20 × 0.17	0.31 × 0.23 × 0.16	0.10 × 0.07 × 0.05
Radiation	MoKα (λ = 0.71073)	MoKα (λ = 0.71073)	MoKα (λ = 0.71073)
2Θ range for data collection/°	5.8 to 50.1	6.46 to 50.08	5.76 to 50.12
Index ranges	-11 ≤ h ≤ 11, -23 ≤ k ≤ 23, -24 ≤ l ≤ 24	-41 ≤ h ≤ 42, -13 ≤ k ≤ 13, -27 ≤ l ≤ 26	-18 ≤ h ≤ 18, -19 ≤ k ≤ 19, -23 ≤ l ≤ 23
Reflections collected	53081	32816	34536
Independent reflections	7217 [R_{int} = 0.0745]	7932 [R_{int} = 0.0414]	15245 [R_{int} = 0.1551]
Data/restraints/parameters	7217/0/534	7932/0/563	15245/0/583
Goodness-of-fit on F^2	1.158	1.101	0.965
Final R indexes [I>=2σ (I)]	R_1 = 0.0582, wR_2 = 0.1068	R_1 = 0.0276, wR_2 = 0.0527	R_1 = 0.0963, wR_2 = 0.1632
Final R indexes [all data]	R_1 = 0.0924, wR_2 = 0.1283	R_1 = 0.0364, wR_2 = 0.0580	R_1 = 0.2156, wR_2 = 0.2540
Largest diff. peak/hole / e Å^{-3}	2.83/-1.47	0.72/-0.66	3.56/-1.46

Table SI2: *Bond Lengths for* **(3).**

Atom	Atom	Length/Å	Atom	Atom	Length/Å
Dy1	Na1[1]	3.324(3)	C26	C27	1.345(12)
Dy1	N11	2.531(7)	C27	C28	1.388(12)
Dy1	O12	2.318(6)	Cl28	C28	1.753(9)
Dy1	O19	2.323(5)	C28	C29	1.388(12)
Dy1	N21	2.591(6)	O29	C29	1.290(9)
Dy1	O29	2.319(6)	C29	C30	1.452(11)
Dy1	N31	2.547(7)	N31	C32	1.329(10)
Dy1	O39	2.282(5)	N31	C40	1.377(10)
Dy1	N41	2.564(7)	C32	C33	1.430(11)
Na1	O12[1]	2.308(7)	C33	C34	1.366(12)
Na1	O19[1]	2.375(6)	C34	C35	1.406(12)
Na1	Cl28[1]	3.018(4)	C35	C36	1.420(12)
Na1	O29[1]	2.307(6)	C35	C40	1.435(11)
Na1	O100	2.175(8)	Cl36	C36	1.754(8)
N11	C12	1.322(10)	C36	C37	1.365(12)
N11	C20	1.380(10)	C37	C38	1.402(12)
O12	C49	1.288(10)	Cl38	C38	1.738(8)
C12	C13	1.423(12)	C38	C39	1.396(11)
C13	C14	1.380(12)	O39	C39	1.292(9)
C14	C15	1.404(12)	C39	C40	1.422(12)
C15	C16	1.421(12)	N41	C42	1.323(11)
C15	C20	1.424(11)	N41	C50	1.398(10)
Cl16	C16	1.753(9)	C42	C43	1.394(12)
C16	C17	1.367(13)	C43	C44	1.420(13)
C17	C18	1.392(12)	C44	C45	1.397(12)
Cl18	C18	1.743(9)	C45	C46	1.426(11)
C18	C19	1.403(11)	C45	C50	1.413(11)
O19	C19	1.303(9)	Cl46	C46	1.734(8)
C19	C20	1.442(12)	C46	C47	1.393(12)
N21	C22	1.340(10)	C47	C48	1.392(12)
N21	C30	1.375(10)	Cl48	C48	1.741(9)
C22	C23	1.405(12)	C48	C49	1.399(11)
C23	C24	1.360(12)	C49	C50	1.426(12)
C24	C25	1.422(12)	O100	C103	1.251(13)
C25	C26	1.396(12)	N100	C101	1.462(13)
C25	C30	1.409(11)	N100	C102	1.436(14)
Cl26	C26	1.754(9)	N100	C103	1.321(13)

[1]1-X,1-Y,2-Z

Table SI3: *Bond Angles for* **(3).**

**Atom**	**Atom**	**Atom**	**Angle/˚**	**Atom**	**Atom**	**Atom**	**Angle/˚**
N11	Dy1	Na1^{1}	94.15(16)	C15	C20	C19	122.5(7)
N11	Dy1	N21	156.1(2)	C22	N21	Dy1	129.2(6)
N11	Dy1	N31	93.7(2)	C22	N21	C30	117.6(7)
N11	Dy1	N41	79.6(2)	C30	N21	Dy1	112.0(5)
O12	Dy1	Na1^{1}	43.95(16)	N21	C22	C23	122.6(8)
O12	Dy1	N11	80.7(2)	C24	C23	C22	120.2(8)
O12	Dy1	O19	77.0(2)	C23	C24	C25	119.3(8)
O12	Dy1	N21	107.5(2)	C26	C25	C24	125.0(8)
O12	Dy1	O29	72.5(2)	C26	C25	C30	117.7(8)
O12	Dy1	N31	150.5(2)	C30	C25	C24	117.2(8)
O12	Dy1	N41	66.5(2)	C25	C26	Cl26	119.7(7)
O19	Dy1	Na1^{1}	45.61(15)	C27	C26	C25	120.2(8)
O19	Dy1	N11	67.3(2)	C27	C26	Cl26	120.1(7)
O19	Dy1	N21	135.8(2)	C26	C27	C28	122.6(9)
O19	Dy1	N31	74.2(2)	C28	Cl28	Na1^{1}	94.9(3)
O19	Dy1	N41	134.0(2)	C27	C28	Cl28	120.2(7)
N21	Dy1	Na1^{1}	107.31(16)	C29	C28	C27	121.7(8)
O29	Dy1	Na1^{1}	43.93(15)	C29	C28	Cl28	118.0(7)
O29	Dy1	N11	137.4(2)	Na1^{1}	O29	Dy1	91.9(2)
O29	Dy1	O19	74.61(19)	C29	O29	Dy1	120.3(5)
O29	Dy1	N21	65.8(2)	C29	O29	Na1^{1}	126.6(5)
O29	Dy1	N31	93.8(2)	C28	C29	C30	114.6(7)
O29	Dy1	N41	117.0(2)	O29	C29	C28	125.3(8)
N31	Dy1	Na1^{1}	108.52(16)	O29	C29	C30	120.0(8)
N31	Dy1	N21	89.3(2)	N21	C30	C25	122.9(7)
N31	Dy1	N41	141.2(2)	N21	C30	C29	114.6(7)
O39	Dy1	Na1^{1}	174.80(15)	C25	C30	C29	122.5(8)
O39	Dy1	N11	84.0(2)	C32	N31	Dy1	128.5(5)
O39	Dy1	O12	140.0(2)	C32	N31	C40	118.4(7)
O39	Dy1	O19	129.40(19)	C40	N31	Dy1	112.3(5)
O39	Dy1	N21	75.5(2)	N31	C32	C33	123.8(8)
O39	Dy1	O29	136.9(2)	C34	C33	C32	117.8(8)
O39	Dy1	N31	66.8(2)	C33	C34	C35	120.9(8)
O39	Dy1	N41	74.5(2)	C34	C35	C36	126.6(8)
N41	Dy1	Na1^{1}	110.02(17)	C34	C35	C40	117.7(8)
N41	Dy1	N21	83.3(2)	C36	C35	C40	115.7(8)
O12^{1}	Na1	Dy1^{1}	44.20(15)	C35	C36	Cl36	118.5(7)
O12^{1}	Na1	O19^{1}	76.2(2)	C37	C36	C35	122.5(8)
O12^{1}	Na1	Cl28^{1}	107.2(2)	C37	C36	Cl36	119.0(6)
O19^{1}	Na1	Dy1^{1}	44.34(14)	C36	C37	C38	119.7(8)
O19^{1}	Na1	Cl28^{1}	137.1(2)	C37	C38	Cl38	118.6(6)

Cl28[1]	Na1	Dy1[1]	108.41(11)	C39	C38	C37	122.8(8)
O29[1]	Na1	Dy1[1]	44.21(15)	C39	C38	Cl38	118.6(6)
O29[1]	Na1	O12[1]	73.0(2)	C39	O39	Dy1	121.5(5)
O29[1]	Na1	O19[1]	73.8(2)	C38	C39	C40	115.8(7)
O29[1]	Na1	Cl28[1]	66.97(17)	O39	C39	C38	123.6(8)
O100	Na1	Dy1[1]	163.1(3)	O39	C39	C40	120.5(7)
O100	Na1	O12[1]	141.1(3)	N31	C40	C35	121.3(8)
O100	Na1	O19[1]	118.9(3)	N31	C40	C39	115.3(7)
O100	Na1	Cl28[1]	85.9(3)	C39	C40	C35	123.4(8)
O100	Na1	O29[1]	143.6(3)	C42	N41	Dy1	128.4(6)
C12	N11	Dy1	128.0(5)	C42	N41	C50	117.8(7)
C12	N11	C20	118.5(7)	C50	N41	Dy1	113.7(5)
C20	N11	Dy1	113.0(5)	N41	C42	C43	126.3(9)
Na1[1]	O12	Dy1	91.8(2)	C42	C43	C44	115.8(9)
C49	O12	Dy1	123.5(5)	C45	C44	C43	120.4(8)
C49	O12	Na1[1]	144.4(5)	C44	C45	C46	124.3(8)
N11	C12	C13	123.5(8)	C44	C45	C50	119.1(8)
C14	C13	C12	117.9(8)	C50	C45	C46	116.6(8)
C13	C14	C15	120.5(8)	C45	C46	Cl46	120.6(7)
C14	C15	C16	125.7(8)	C47	C46	C45	120.6(8)
C14	C15	C20	117.6(8)	C47	C46	Cl46	118.8(6)
C16	C15	C20	116.6(8)	C48	C47	C46	120.2(8)
C15	C16	Cl16	118.1(7)	C47	C48	Cl48	118.9(6)
C17	C16	C15	122.5(8)	C47	C48	C49	123.2(8)
C17	C16	Cl16	119.4(7)	C49	C48	Cl48	117.9(7)
C16	C17	C18	119.2(8)	O12	C49	C48	124.0(8)
C17	C18	Cl18	118.2(7)	O12	C49	C50	120.9(7)
C17	C18	C19	123.7(8)	C48	C49	C50	115.1(8)
C19	C18	Cl18	118.1(7)	N41	C50	C45	120.6(8)
Dy1	O19	Na1[1]	90.05(19)	N41	C50	C49	115.1(7)
C19	O19	Dy1	120.4(5)	C45	C50	C49	124.3(7)
C19	O19	Na1[1]	129.8(5)	C103	O100	Na1	148.4(8)
C18	C19	C20	115.3(8)	C102	N100	C101	118.5(10)
O19	C19	C18	124.8(8)	C103	N100	C101	118.3(10)
O19	C19	C20	119.9(7)	C103	N100	C102	122.8(10)
N11	C20	C15	121.7(8)	O100	C103	N100	122.8(12)
N11	C20	C19	115.9(7)				

[1]1-X,1-Y,2-Z

Table SI4: *Bond Lengths for* **(4).**

Atom	Atom	Length/Å	Atom	Atom	Length/Å
Dy1	N11	2.577(3)	N31	C32	1.325(4)
Dy1	O19	2.295(2)	N31	C40	1.364(4)
Dy1	N21	2.634(2)	C32	C33	1.403(4)
Dy1	O29	2.269(2)	C33	C34	1.364(5)
Dy1	N31	2.672(3)	C34	C35	1.417(4)
Dy1	O39	2.287(2)	C35	C36	1.420(5)
Dy1	N41	2.519(2)	C35	C40	1.425(4)
Dy1	O49	2.292(2)	C36	Cl36	1.751(3)
N11	C12	1.326(4)	C36	C37	1.355(4)
N11	C20	1.375(4)	C37	C38	1.410(4)
C12	C13	1.427(4)	C38	Cl38	1.742(3)
C13	C14	1.359(4)	C38	C39	1.390(4)
C14	C15	1.416(4)	O39	C39	1.299(3)
C15	C16	1.420(4)	C39	C40	1.446(4)
C15	C20	1.421(4)	N41	C42	1.331(4)
C16	Cl16	1.748(3)	N41	C50	1.365(4)
C16	C17	1.360(4)	C42	C43	1.394(4)
C17	C18	1.400(4)	C43	C44	1.370(4)
C18	Cl18	1.746(3)	C44	C45	1.405(4)
C18	C19	1.397(4)	C45	C46	1.413(4)
O19	C19	1.297(4)	C45	C50	1.414(4)
C19	C20	1.444(4)	C46	Cl46	1.743(3)
N21	C22	1.325(4)	C46	C47	1.365(4)
N21	C30	1.379(4)	C47	C48	1.408(4)
C22	C23	1.410(4)	C48	Cl48	1.745(3)
C23	C24	1.366(4)	C48	C49	1.390(4)
C24	C25	1.410(4)	O49	C49	1.302(3)
C25	C26	1.417(4)	C49	C50	1.457(4)
C25	C30	1.427(4)	N100	C101	1.522(4)
C26	Cl26	1.750(3)	N100	C111	1.519(4)
C26	C27	1.365(4)	N100	C121	1.520(4)
C27	C28	1.403(4)	N100	C131	1.514(4)
C28	Cl28	1.742(3)	C101	C102	1.511(4)
C28	C29	1.387(4)	C111	C112	1.510(5)
O29	C29	1.298(3)	C121	C122	1.519(4)
C29	C30	1.434(4)	C131	C132	1.516(5)

Table SI5: *Bond Angles for* **(4).**

Atom	Atom	Atom	Angle/˚	Atom	Atom	Atom	Angle/˚
N11	Dy1	N21	138.40(8)	C29	C28	C27	123.1(3)
N11	Dy1	N31	73.50(8)	C29	C28	Cl28	117.1(2)
O19	Dy1	N11	66.17(8)	C29	O29	Dy1	125.75(19)
O19	Dy1	N21	153.88(8)	C28	C29	C30	115.4(3)
O19	Dy1	N31	87.46(7)	O29	C29	C28	124.0(3)
O19	Dy1	N41	79.46(8)	O29	C29	C30	120.5(3)
N21	Dy1	N31	106.58(7)	N21	C30	C25	122.0(3)
O29	Dy1	N11	74.10(8)	N21	C30	C29	115.3(3)
O29	Dy1	O19	139.94(7)	C25	C30	C29	122.8(3)
O29	Dy1	N21	65.95(7)	C32	N31	Dy1	129.4(2)
O29	Dy1	N31	76.37(8)	C32	N31	C40	117.7(3)
O29	Dy1	O39	114.53(7)	C40	N31	Dy1	112.8(2)
O29	Dy1	N41	136.18(7)	N31	C32	C33	123.9(3)
O29	Dy1	O49	85.37(7)	C34	C33	C32	118.8(3)
O39	Dy1	N11	132.19(8)	C33	C34	C35	120.2(3)
O39	Dy1	O19	89.64(7)	C34	C35	C36	125.9(3)
O39	Dy1	N21	77.44(7)	C34	C35	C40	116.5(3)
O39	Dy1	N31	64.42(8)	C36	C35	C40	117.6(3)
O39	Dy1	N41	73.80(8)	C35	C36	Cl36	119.6(3)
O39	Dy1	O49	138.48(7)	C37	C36	C35	121.4(3)
N41	Dy1	N11	134.15(8)	C37	C36	Cl36	119.0(3)
N41	Dy1	N21	75.18(8)	C36	C37	C38	120.2(3)
N41	Dy1	N31	136.19(8)	C37	C38	Cl38	119.0(3)
O49	Dy1	N11	87.12(7)	C39	C38	C37	123.1(3)
O49	Dy1	O19	97.27(7)	C39	C38	Cl38	117.9(3)
O49	Dy1	N21	78.82(7)	C39	O39	Dy1	126.72(19)
O49	Dy1	N31	156.28(7)	C38	C39	C40	115.7(3)
O49	Dy1	N41	67.44(7)	O39	C39	C38	125.0(3)
C12	N11	Dy1	127.8(2)	O39	C39	C40	119.3(3)
C12	N11	C20	117.7(3)	N31	C40	C35	122.8(3)
C20	N11	Dy1	112.94(19)	N31	C40	C39	115.1(3)
N11	C12	C13	124.1(3)	C35	C40	C39	122.0(3)
C14	C13	C12	117.7(3)	C42	N41	Dy1	127.4(2)
C13	C14	C15	121.1(3)	C42	N41	C50	117.8(3)
C14	C15	C16	125.9(3)	C50	N41	Dy1	114.77(19)
C14	C15	C20	117.0(3)	N41	C42	C43	123.8(3)
C16	C15	C20	117.1(3)	C44	C43	C42	118.6(3)
C15	C16	Cl16	118.8(3)	C43	C44	C45	120.2(3)
C17	C16	C15	121.3(3)	C44	C45	C46	125.1(3)
C17	C16	Cl16	119.9(3)	C44	C45	C50	117.3(3)
C16	C17	C18	120.4(3)	C46	C45	C50	117.6(3)

C17	C18	Cl18	118.3(2)	C45	C46	Cl46	119.6(3)
C19	C18	C17	123.3(3)	C47	C46	C45	121.4(3)
C19	C18	Cl18	118.4(3)	C47	C46	Cl46	119.0(3)
C19	O19	Dy1	123.17(19)	C46	C47	C48	120.0(3)
C18	C19	C20	114.8(3)	C47	C48	Cl48	117.3(2)
O19	C19	C18	125.1(3)	C49	C48	C47	123.3(3)
O19	C19	C20	120.1(3)	C49	C48	Cl48	119.3(2)
N11	C20	C15	122.4(3)	C49	O49	Dy1	122.50(19)
N11	C20	C19	114.6(3)	C48	C49	C50	114.9(3)
C15	C20	C19	123.0(3)	O49	C49	C48	125.6(3)
C22	N21	Dy1	129.4(2)	O49	C49	C50	119.5(3)
C22	N21	C30	118.2(3)	N41	C50	C45	122.4(3)
C30	N21	Dy1	112.39(19)	N41	C50	C49	115.0(3)
N21	C22	C23	123.2(3)	C45	C50	C49	122.6(3)
C24	C23	C22	119.3(3)	C111	N100	C101	109.0(3)
C23	C24	C25	120.0(3)	C111	N100	C121	111.4(3)
C24	C25	C26	125.5(3)	C121	N100	C101	107.6(2)
C24	C25	C30	117.3(3)	C131	N100	C101	111.5(3)
C26	C25	C30	117.2(3)	C131	N100	C111	108.6(3)
C25	C26	Cl26	119.4(2)	C131	N100	C121	108.7(2)
C27	C26	C25	120.8(3)	C102	C101	N100	115.4(3)
C27	C26	Cl26	119.8(2)	C112	C111	N100	116.5(3)
C26	C27	C28	120.5(3)	C122	C121	N100	115.4(3)
C27	C28	Cl28	119.8(2)	N100	C131	C132	115.6(3)

Table SI6: *Bond Lengths for* **(5).**

**Atom**	**Atom**	**Length/Å**	**Atom**	**Atom**	**Length/Å**
Dy1	N11	2.525(10)	O49	C49	1.300(15)
Dy1	O19	2.305(9)	O49	K1[1]	3.048(11)
Dy1	N21	2.559(12)	C49	C50	1.451(19)
Dy1	O29	2.318(11)	N51	C52	1.321(19)
Dy1	N31	2.537(11)	N51	C60	1.389(16)
Dy1	O39	2.314(9)	C52	C53	1.39(2)
Dy1	N41	2.601(12)	C53	C54	1.376(19)
Dy1	O49	2.275(9)	C54	C55	1.39(2)
Dy1	K1[1]	3.9634(9)	C55	C56	1.413(18)
Dy2	N51	2.526(13)	C55	C60	1.427(19)
Dy2	O59	2.325(9)	C56	Cl56	1.748(16)
Dy2	N61	2.550(11)	C56	C57	1.37(2)

Dy2	O69	2.300(10)	C57	C58	1.42(2)
Dy2	N71	2.591(13)	C58	Cl58	1.705(15)
Dy2	O79	2.286(10)	C58	C59	1.368(18)
Dy2	N81	2.564(11)	O59	C59	1.299(16)
Dy2	O89	2.333(9)	C59	C60	1.45(2)
Dy2	K2	3.9783(9)	N61	C62	1.320(16)
N11	C12	1.301(18)	N61	C70	1.346(17)
N11	C20	1.404(17)	C62	C63	1.418(16)
C12	C13	1.391(19)	C63	C64	1.350(18)
C13	C14	1.341(19)	C64	C65	1.378(19)
C14	C15	1.39(2)	C65	C66	1.384(19)
C15	C16	1.43(2)	C65	C70	1.406(17)
C15	C20	1.427(19)	C66	Cl66	1.725(13)
C16	Cl16	1.729(14)	C66	C67	1.435(19)
C16	C17	1.37(2)	C67	C68	1.392(18)
C17	C18	1.374(18)	C68	Cl68	1.739(15)
C18	Cl18	1.747(16)	C68	C69	1.40(2)
C18	C19	1.419(19)	Cl68	K2	3.698(4)
O19	C19	1.280(15)	O69	C69	1.307(14)
C19	C20	1.43(2)	O69	K2	2.910(8)
N21	C22	1.312(17)	C69	C70	1.477(18)
N21	C30	1.350(18)	N71	C72	1.296(16)
C22	C23	1.450(18)	N71	C80	1.389(17)
C23	C24	1.40(2)	C72	C73	1.44(2)
C24	C25	1.39(2)	C73	C74	1.365(19)
C25	C26	1.41(2)	C74	C75	1.407(19)
C25	C30	1.419(18)	C75	C76	1.43(2)
C26	Cl26	1.760(14)	C75	C80	1.40(2)
C26	C27	1.36(2)	C76	Cl76	1.733(18)
C27	C28	1.392(18)	C76	C77	1.38(2)
C28	Cl28	1.733(15)	C77	C78	1.40(2)
C28	C29	1.42(2)	C78	Cl78	1.756(15)
Cl28	K1[1]	3.400(4)	C78	C79	1.37(2)
O29	C29	1.301(15)	O79	C79	1.307(18)
O29	K1[1]	2.821(10)	O79	K2	3.247(10)
C29	C30	1.40(2)	C79	C80	1.399(19)
N31	C32	1.325(18)	N81	C82	1.314(16)
N31	C40	1.392(16)	N81	C90	1.388(16)
C32	C33	1.412(19)	C82	C83	1.384(17)
C33	C34	1.392(19)	C83	C84	1.386(17)
C34	C35	1.41(2)	C84	C85	1.423(19)
C35	C36	1.42(2)	C85	C86	1.436(18)
C35	C40	1.417(19)	C85	C90	1.435(19)

C36	Cl36	1.752(14)	C86	Cl86	1.737(13)
C36	C37	1.38(2)	C86	C87	1.363(18)
C37	C38	1.390(19)	C87	C88	1.413(17)
C38	Cl38	1.761(16)	C88	Cl88	1.728(14)
C38	C39	1.380(18)	C88	C89	1.375(17)
C39	O39	1.304(15)	Cl88	K2	3.436(4)
C39	C40	1.43(2)	O89	C89	1.298(14)
O39	K1[1]	2.965(9)	O89	K2	2.790(9)
N41	C42	1.284(17)	C89	C90	1.417(19)
N41	C50	1.373(15)	N101	C101	1.49(2)
C42	C43	1.387(19)	N101	C103	1.51(2)
C43	C44	1.374(17)	N101	C105	1.512(16)
C44	C45	1.357(18)	N101	C107	1.545(18)
C45	C46	1.412(18)	C101	C102	1.61(2)
C45	C50	1.435(19)	C103	C104	1.550(18)
C46	Cl46	1.760(16)	C105	C106	1.53(2)
C46	C47	1.39(2)	C107	C108	1.60(2)
C47	C48	1.375(18)	N201	C201	1.144(18)
C48	Cl48	1.763(14)	C201	C202	1.50(2)
C48	C49	1.402(16)	C301	N301	1.13(2)
Cl48	K1[1]	3.725(4)	C301	C302	1.44(2)

[1]1+X,-1+Y,+Z

Table SI7: *Bond Angles for* **(5).**

**Atom**	**Atom**	**Atom**	**Angle/˚**	**Atom**	**Atom**	**Atom**	**Angle/˚**
N11	Dy1	N21	93.8(3)	C67	C68	Cl68	118.9(13)
N11	Dy1	N31	95.9(3)	C67	C68	C69	122.1(14)
N11	Dy1	N41	142.7(4)	C69	C68	Cl68	118.9(11)
N11	Dy1	K1[1]	100.5(3)	C68	Cl68	K2	95.6(5)
O19	Dy1	N11	67.2(4)	Dy2	O69	K2	98.9(3)
O19	Dy1	N21	74.1(4)	C69	O69	Dy2	122.8(9)
O19	Dy1	O29	125.2(3)	C69	O69	K2	125.8(8)
O19	Dy1	N31	81.8(4)	C68	C69	C70	116.2(12)
O19	Dy1	O39	128.7(3)	O69	C69	C68	123.2(13)
O19	Dy1	N41	75.5(4)	O69	C69	C70	120.6(13)
O19	Dy1	K1[1]	166.8(3)	N61	C70	C65	125.6(14)
N21	Dy1	N41	76.2(4)	N61	C70	C69	113.1(12)
N21	Dy1	K1[1]	103.0(3)	C65	C70	C69	121.2(14)

O29	Dy1	N11	79.5(3)	C72	N71	Dy2	127.7(11)
O29	Dy1	N21	65.9(4)	C72	N71	C80	120.4(14)
O29	Dy1	N31	145.9(4)	C80	N71	Dy2	111.8(9)
O29	Dy1	N41	125.4(3)	N71	C72	C73	122.4(15)
O29	Dy1	K1[1]	44.4(2)	C74	C73	C72	118.5(15)
N31	Dy1	N21	148.1(4)	C73	C74	C75	119.2(17)
N31	Dy1	N41	77.7(4)	C74	C75	C76	122.7(16)
N31	Dy1	K1[1]	105.0(3)	C80	C75	C74	119.7(15)
O39	Dy1	N11	76.1(3)	C80	C75	C76	117.4(14)
O39	Dy1	N21	144.7(4)	C75	C76	Cl76	120.7(13)
O39	Dy1	O29	79.0(3)	C77	C76	C75	118.1(17)
O39	Dy1	N31	67.3(4)	C77	C76	Cl76	121.0(13)
O39	Dy1	N41	130.9(4)	C76	C77	C78	122.0(16)
O39	Dy1	K1[1]	48.0(2)	C77	C78	Cl78	117.6(13)
N41	Dy1	K1[1]	116.7(3)	C79	C78	C77	121.5(16)
O49	Dy1	N11	150.4(4)	C79	C78	Cl78	120.7(14)
O49	Dy1	O19	142.3(4)	Dy2	O79	K2	90.2(3)
O49	Dy1	N21	94.5(3)	C79	O79	Dy2	122.2(9)
O49	Dy1	O29	78.3(3)	C79	O79	K2	147.3(9)
O49	Dy1	N31	91.8(3)	C78	C79	C80	116.4(16)
O49	Dy1	O39	80.7(3)	O79	C79	C78	120.9(15)
O49	Dy1	N41	66.8(4)	O79	C79	C80	122.6(14)
O49	Dy1	K1[1]	50.0(3)	N71	C80	C75	119.5(14)
N51	Dy2	N61	99.6(4)	N71	C80	C79	116.0(15)
N51	Dy2	N71	142.7(4)	C79	C80	C75	124.4(15)
N51	Dy2	N81	89.5(4)	C82	N81	Dy2	127.5(9)
N51	Dy2	K2	95.6(3)	C82	N81	C90	118.2(12)
O59	Dy2	N51	67.1(4)	C90	N81	Dy2	114.2(9)
O59	Dy2	N61	84.0(3)	N81	C82	C83	125.7(13)
O59	Dy2	N71	75.6(4)	C82	C83	C84	119.2(15)
O59	Dy2	N81	72.0(3)	C83	C84	C85	117.1(14)
O59	Dy2	O89	125.7(3)	C84	C85	C86	124.0(14)
O59	Dy2	K2	162.3(2)	C84	C85	C90	120.3(13)
N61	Dy2	N71	76.2(4)	C90	C85	C86	115.7(14)
N61	Dy2	N81	148.6(4)	C85	C86	Cl86	117.7(11)
N61	Dy2	K2	102.9(3)	C87	C86	C85	120.0(13)
O69	Dy2	N51	74.7(4)	C87	C86	Cl86	122.2(10)
O69	Dy2	O59	126.4(3)	C86	C87	C88	121.7(13)
O69	Dy2	N61	66.6(4)	C87	C88	Cl88	119.3(11)
O69	Dy2	N71	132.2(4)	C89	C88	C87	122.1(14)
O69	Dy2	N81	144.5(3)	C89	C88	Cl88	118.3(11)
O69	Dy2	O89	79.9(3)	C88	Cl88	K2	100.2(5)
O69	Dy2	K2	46.3(2)	Dy2	O89	K2	101.5(3)

N71	Dy2	K2	121.7(3)	C89	O89	Dy2	123.7(9)
O79	Dy2	N51	149.9(4)	C89	O89	K2	132.1(8)
O79	Dy2	O59	142.9(4)	C88	C89	C90	115.6(13)
O79	Dy2	N61	84.7(3)	O89	C89	C88	124.4(13)
O79	Dy2	O69	79.9(4)	O89	C89	C90	119.6(12)
O79	Dy2	N71	67.3(4)	N81	C90	C85	119.2(14)
O79	Dy2	N81	102.3(3)	N81	C90	C89	115.9(13)
O79	Dy2	O89	80.2(3)	C89	C90	C85	124.4(13)
O79	Dy2	K2	54.7(3)	Dy1^2	K1	Dy1^3	180.0
N81	Dy2	N71	78.4(4)	Cl28^2	K1	Dy1^2	91.40(7)
N81	Dy2	K2	106.1(2)	Cl28^3	K1	Dy1^2	88.60(7)
O89	Dy2	N51	79.7(4)	Cl28^3	K1	Dy1^3	91.40(7)
O89	Dy2	N61	145.2(4)	Cl28^2	K1	Dy1^3	88.60(7)
O89	Dy2	N71	124.6(3)	Cl28^3	K1	Cl28^2	180.0
O89	Dy2	N81	65.9(3)	Cl28^3	K1	Cl48^2	60.55(9)
O89	Dy2	K2	43.4(2)	Cl28^2	K1	Cl48^2	119.45(9)
C12	N11	Dy1	127.5(10)	Cl28^3	K1	Cl48^3	119.45(9)
C12	N11	C20	119.3(13)	Cl28^2	K1	Cl48^3	60.55(9)
C20	N11	Dy1	112.7(9)	O29^3	K1	Dy1^2	144.9(2)
N11	C12	C13	122.2(15)	O29^3	K1	Dy1^3	35.1(2)
C14	C13	C12	120.6(16)	O29^2	K1	Dy1^3	144.9(2)
C13	C14	C15	120.2(15)	O29^2	K1	Dy1^2	35.1(2)
C14	C15	C16	126.3(14)	O29^3	K1	Cl28^3	56.3(2)
C14	C15	C20	117.7(15)	O29^2	K1	Cl28^2	56.3(2)
C20	C15	C16	116.0(15)	O29^2	K1	Cl28^3	123.7(2)
C15	C16	Cl16	120.4(12)	O29^3	K1	Cl28^2	123.7(2)
C17	C16	C15	119.7(14)	O29^2	K1	O29^3	180.0
C17	C16	Cl16	119.9(12)	O29^2	K1	O39^2	61.1(3)
C16	C17	C18	122.5(15)	O29^3	K1	O39^2	118.9(3)
C17	C18	Cl18	120.2(12)	O29^3	K1	O39^3	61.1(3)
C17	C18	C19	123.5(15)	O29^2	K1	O39^3	118.9(3)
C19	C18	Cl18	116.3(11)	O29^2	K1	Cl48^2	101.9(2)
C19	O19	Dy1	121.2(10)	O29^2	K1	Cl48^3	78.1(2)
C18	C19	C20	112.7(14)	O29^3	K1	Cl48^2	78.1(2)
O19	C19	C18	126.8(15)	O29^3	K1	Cl48^3	101.9(2)
O19	C19	C20	120.5(14)	O29^3	K1	O49^2	121.0(3)
N11	C20	C15	119.6(15)	O29^2	K1	O49^3	121.0(3)
N11	C20	C19	114.8(13)	O29^2	K1	O49^2	59.0(3)
C15	C20	C19	125.6(15)	O29^3	K1	O49^3	59.0(3)
C22	N21	Dy1	125.8(11)	O39^2	K1	Dy1^2	35.46(18)
C22	N21	C30	119.8(13)	O39^3	K1	Dy1^3	35.46(18)
C30	N21	Dy1	114.2(10)	O39^3	K1	Dy1^2	144.54(18)
N21	C22	C23	125.4(15)	O39^2	K1	Dy1^3	144.54(18)

C24	C23	C22	112.8(16)	O39[2]	K1	Cl28[2]	108.7(2)
C25	C24	C23	123.2(15)	O39[3]	K1	Cl28[2]	71.3(2)
C24	C25	C26	124.5(15)	O39[3]	K1	Cl28[3]	108.7(2)
C24	C25	C30	118.1(16)	O39[2]	K1	Cl28[3]	71.3(2)
C26	C25	C30	117.3(16)	O39[2]	K1	O39[3]	180.0
C25	C26	Cl26	119.4(13)	O39[2]	K1	Cl48[2]	103.1(2)
C27	C26	C25	120.9(15)	O39[3]	K1	Cl48[2]	76.9(2)
C27	C26	Cl26	119.6(13)	O39[2]	K1	Cl48[3]	76.9(2)
C26	C27	C28	121.0(17)	O39[3]	K1	Cl48[3]	103.1(2)
C27	C28	Cl28	120.7(13)	O39[2]	K1	O49[2]	59.2(2)
C27	C28	C29	121.5(16)	O39[2]	K1	O49[3]	120.8(2)
C29	C28	Cl28	117.7(11)	O39[3]	K1	O49[2]	120.8(2)
C28	Cl28	K1[1]	95.9(5)	O39[3]	K1	O49[3]	59.2(2)
Dy1	O29	K1[1]	100.5(3)	Cl48[2]	K1	Dy1[3]	93.99(7)
C29	O29	Dy1	122.3(10)	Cl48[2]	K1	Dy1[2]	86.01(7)
C29	O29	K1[1]	120.4(8)	Cl48[3]	K1	Dy1[2]	93.99(7)
O29	C29	C28	123.5(15)	Cl48[3]	K1	Dy1[3]	86.01(7)
O29	C29	C30	120.8(15)	Cl48[2]	K1	Cl48[3]	180.0
C30	C29	C28	115.7(13)	O49[3]	K1	Dy1[2]	145.14(16)
N21	C30	C25	120.6(16)	O49[3]	K1	Dy1[3]	34.86(16)
N21	C30	C29	115.9(14)	O49[2]	K1	Dy1[2]	34.86(16)
C29	C30	C25	123.5(16)	O49[2]	K1	Dy1[3]	145.14(16)
C32	N31	Dy1	128.9(10)	O49[3]	K1	Cl28[3]	108.14(19)
C32	N31	C40	116.8(13)	O49[2]	K1	Cl28[2]	108.14(19)
C40	N31	Dy1	114.2(9)	O49[3]	K1	Cl28[2]	71.86(19)
N31	C32	C33	126.6(15)	O49[2]	K1	Cl28[3]	71.86(19)
C34	C33	C32	115.8(16)	O49[2]	K1	Cl48[2]	51.16(17)
C33	C34	C35	120.9(16)	O49[3]	K1	Cl48[3]	51.16(17)
C34	C35	C36	123.7(15)	O49[2]	K1	Cl48[3]	128.84(17)
C34	C35	C40	118.1(15)	O49[3]	K1	Cl48[2]	128.84(17)
C40	C35	C36	118.1(16)	O49[2]	K1	O49[3]	180.0
C35	C36	Cl36	119.4(13)	Dy2[4]	K2	Dy2	180.0
C37	C36	C35	119.7(15)	Cl68[4]	K2	Dy2[4]	86.73(6)
C37	C36	Cl36	120.9(12)	Cl68	K2	Dy2[4]	93.27(6)
C36	C37	C38	119.5(15)	Cl68[4]	K2	Dy2	93.27(6)
C37	C38	Cl38	117.3(12)	Cl68	K2	Dy2	86.73(6)
C39	C38	C37	125.4(16)	Cl68[4]	K2	Cl68	180.00(10)
C39	C38	Cl38	117.1(12)	O69	K2	Dy2[4]	145.17(19)
C38	C39	C40	113.9(13)	O69[4]	K2	Dy2[4]	34.83(19)
O39	C39	C38	125.3(15)	O69[4]	K2	Dy2	145.17(19)
O39	C39	C40	120.7(14)	O69	K2	Dy2	34.83(19)
Dy1	O39	K1[1]	96.5(3)	O69	K2	Cl68[4]	127.8(2)
C39	O39	Dy1	122.5(10)	O69[4]	K2	Cl68	127.8(2)

C39	O39	$K1^{1}$	123.8(8)	O69	K2	Cl68	52.2(2)
N31	C40	C35	121.7(14)	$O69^{4}$	K2	$Cl68^{4}$	52.2(2)
N31	C40	C39	115.2(13)	$O69^{4}$	K2	O69	180.0
C35	C40	C39	123.1(14)	$O69^{4}$	K2	$O79^{4}$	56.8(3)
C42	N41	Dy1	128.6(9)	O69	K2	O79	56.8(3)
C42	N41	C50	117.8(13)	$O69^{4}$	K2	O79	123.2(3)
C50	N41	Dy1	113.4(10)	O69	K2	$O79^{4}$	123.2(3)
N41	C42	C43	125.6(14)	$O69^{4}$	K2	$Cl88^{4}$	108.97(19)
C44	C43	C42	117.2(15)	O69	K2	$Cl88^{4}$	71.03(19)
C45	C44	C43	120.8(14)	$O69^{4}$	K2	Cl88	71.03(19)
C44	C45	C46	127.8(14)	O69	K2	Cl88	108.97(19)
C44	C45	C50	117.9(13)	O79	K2	$Dy2^{4}$	144.93(17)
C46	C45	C50	114.3(14)	$O79^{4}$	K2	$Dy2^{4}$	35.07(17)
C45	C46	Cl46	118.3(13)	O79	K2	Dy2	35.07(17)
C47	C46	C45	123.3(15)	$O79^{4}$	K2	Dy2	144.93(17)
C47	C46	Cl46	118.4(11)	$O79^{4}$	K2	Cl68	76.66(17)
C48	C47	C46	119.0(13)	O79	K2	Cl68	103.34(17)
C47	C48	Cl48	118.2(10)	O79	K2	$Cl68^{4}$	76.66(17)
C47	C48	C49	125.1(14)	$O79^{4}$	K2	$Cl68^{4}$	103.34(17)
C49	C48	Cl48	116.6(11)	$O79^{4}$	K2	O79	180.0(4)
C48	Cl48	$K1^{1}$	106.0(4)	O79	K2	$Cl88^{4}$	70.95(18)
Dy1	O49	$K1^{1}$	95.2(3)	O79	K2	Cl88	109.05(18)
C49	O49	Dy1	123.8(9)	$O79^{4}$	K2	$Cl88^{4}$	109.05(18)
C49	O49	$K1^{1}$	140.3(8)	$O79^{4}$	K2	Cl88	70.95(18)
C48	C49	C50	113.0(13)	Cl88	K2	Dy2	90.49(6)
O49	C49	C48	125.3(14)	$Cl88^{4}$	K2	$Dy2^{4}$	90.49(6)
O49	C49	C50	121.6(12)	$Cl88^{4}$	K2	Dy2	89.51(6)
N41	C50	C45	120.6(14)	Cl88	K2	$Dy2^{4}$	89.51(6)
N41	C50	C49	114.2(13)	$Cl88^{4}$	K2	Cl68	62.72(10)
C45	C50	C49	125.2(12)	Cl88	K2	$Cl68^{4}$	62.72(10)
C52	N51	Dy2	127.9(11)	$Cl88^{4}$	K2	$Cl68^{4}$	117.28(10)
C52	N51	C60	117.5(14)	Cl88	K2	Cl68	117.28(10)
C60	N51	Dy2	114.2(10)	$Cl88^{4}$	K2	Cl88	180.0
N51	C52	C53	122.4(16)	O89	K2	$Dy2^{4}$	144.93(18)
C54	C53	C52	120.2(18)	$O89^{4}$	K2	Dy2	144.93(18)
C53	C54	C55	120.6(17)	O89	K2	Dy2	35.07(18)
C54	C55	C56	126.5(15)	$O89^{4}$	K2	$Dy2^{4}$	35.07(18)
C54	C55	C60	115.4(14)	O89	K2	$Cl68^{4}$	73.8(2)
C56	C55	C60	118.0(14)	$O89^{4}$	K2	Cl68	73.8(2)
C55	C56	Cl56	119.3(12)	O89	K2	Cl68	106.2(2)
C57	C56	C55	120.1(15)	$O89^{4}$	K2	$Cl68^{4}$	106.2(2)
C57	C56	Cl56	120.6(12)	$O89^{4}$	K2	O69	117.1(3)
C56	C57	C58	121.8(15)	$O89^{4}$	K2	$O69^{4}$	62.9(3)

C57	C58	Cl58	117.3(11)	O89	K2	O69[4]	117.1(3)
C59	C58	C57	120.5(15)	O89	K2	O69	62.9(3)
C59	C58	Cl58	122.2(12)	O89	K2	O79	58.5(3)
C59	O59	Dy2	122.0(9)	O89	K2	O79[4]	121.5(3)
C58	C59	C60	118.1(14)	O89[4]	K2	O79[4]	58.5(3)
O59	C59	C58	122.4(14)	O89[4]	K2	O79	121.5(3)
O59	C59	C60	119.5(13)	O89	K2	Cl88[4]	124.3(2)
N51	C60	C55	123.4(14)	O89[4]	K2	Cl88[4]	55.7(2)
N51	C60	C59	115.3(14)	O89[4]	K2	Cl88	124.3(2)
C55	C60	C59	121.3(13)	O89	K2	Cl88	55.7(2)
C62	N61	Dy2	128.1(10)	O89[4]	K2	O89	180.0(3)
C62	N61	C70	115.4(12)	C101	N101	C103	117.2(13)
C70	N61	Dy2	116.5(9)	C101	N101	C105	112.9(12)
N61	C62	C63	123.5(14)	C101	N101	C107	101.0(12)
C64	C63	C62	118.8(14)	C103	N101	C105	107.0(13)
C63	C64	C65	120.4(13)	C103	N101	C107	110.1(12)
C64	C65	C66	123.7(13)	C105	N101	C107	108.3(12)
C64	C65	C70	116.0(15)	N101	C101	C102	105.5(14)
C66	C65	C70	120.3(14)	N101	C103	C104	113.9(14)
C65	C66	Cl66	122.0(11)	N101	C105	C106	116.3(14)
C65	C66	C67	119.3(13)	N101	C107	C108	114.7(14)
C67	C66	Cl66	118.6(12)	N201	C201	C202	176(2)
C68	C67	C66	120.8(15)	N301	C301	C302	178(2)

[1]1+X,-1+Y,+Z; [2]3-X,-1-Y,2-Z; [3]-1+X,1+Y,+Z; [4]3-X,-Y,1-Z

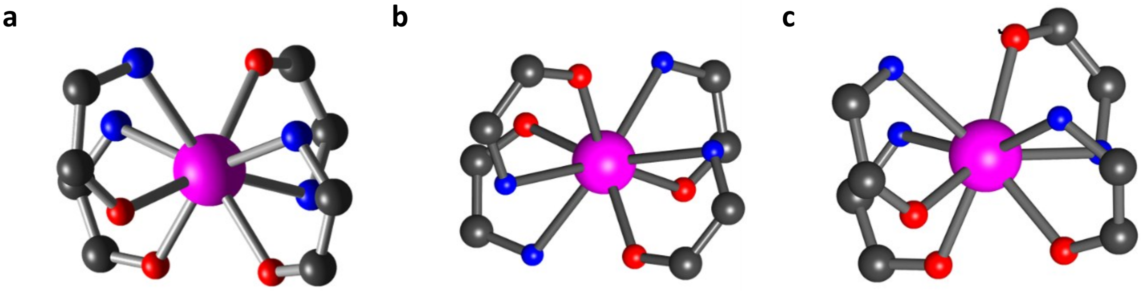

Figure SI2: *First coordination sphere of the* $[Ln(Cl_2q)_4]^-$ based *complexes. a)* *Na[Dy(5,7Cl₂q)₄]*(3) *b)* *NEt₄[Dy(5,7Cl₂q)₄]* (4) *c)* *K₀.₅(NEt₄)₀.₅[Dy(5,7Cl₂q)₄]* (5). *Interestingly, NaDyClq* (3) *and KNetDyClq* (5) *have a similar coordination sphere, but NEtDyClq* (4) *is different since the ligands have a different arrangement around the lanthanide center.*

2. IR spectra

The IR spectra of the powdered compounds are compared with the films finding an excellent agreement. The characteristic vibration modes of the molecules place in the range [1700 - 500] cm^{-1}. The bands in the range [1600 - 1300] cm^{-1} are mainly attributed to C = N and C = C vibration frequencies (ring stretching) while the band at 1100 cm^{-1} is related to the CO stretching.[1,2]. The C - Cl vibration locates in the range [958 - 954] cm^{-1}.

a

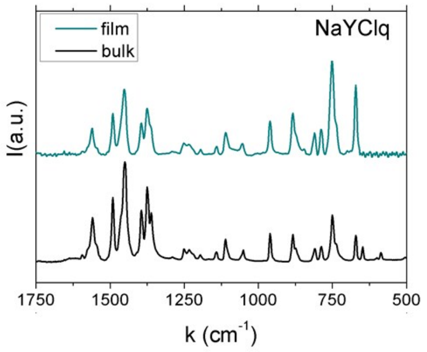

b

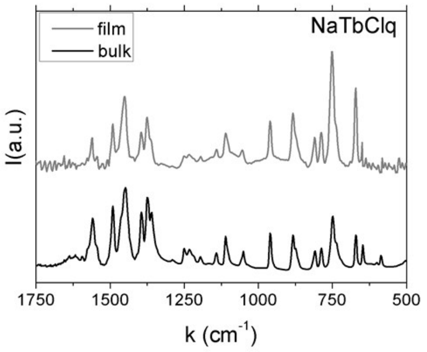

c

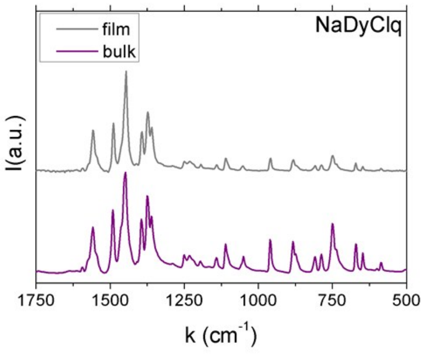

d

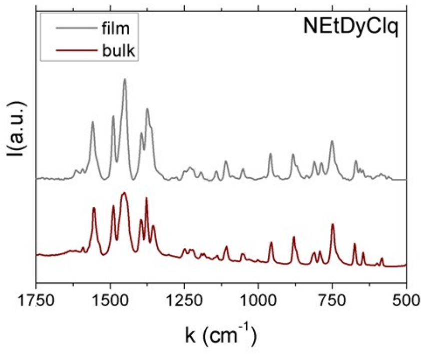

e

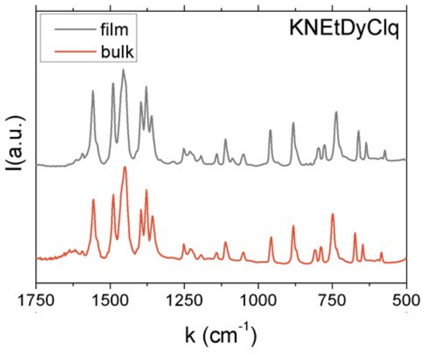

Fig. SI3: IR *transmission spectra for the bulk compounds, NaYClq* (1), *NaTbClq* (2), *NaDyClq* (3), *NEtDyClq* (4) and *KNEtDyClq* (5), *compared with the deposited films where the ligand vibrations can be detected.*

3. Mass Spectrometry

Electrospray ionization mass spectrometry (ESI-MS) has been performed for all bulk compounds. For the five cases the main signal corresponds to the relation mass/charge (m/z) of the ionized molecule: $[Dy[(5,7Cl_2q)_4]]^-$. On the right hand side of the figure the pattern of the main signal is compared to the theoretical pattern, matching in all cases. The secondary signals are attributed to fragmentations of the molecules caused by the technique itself.

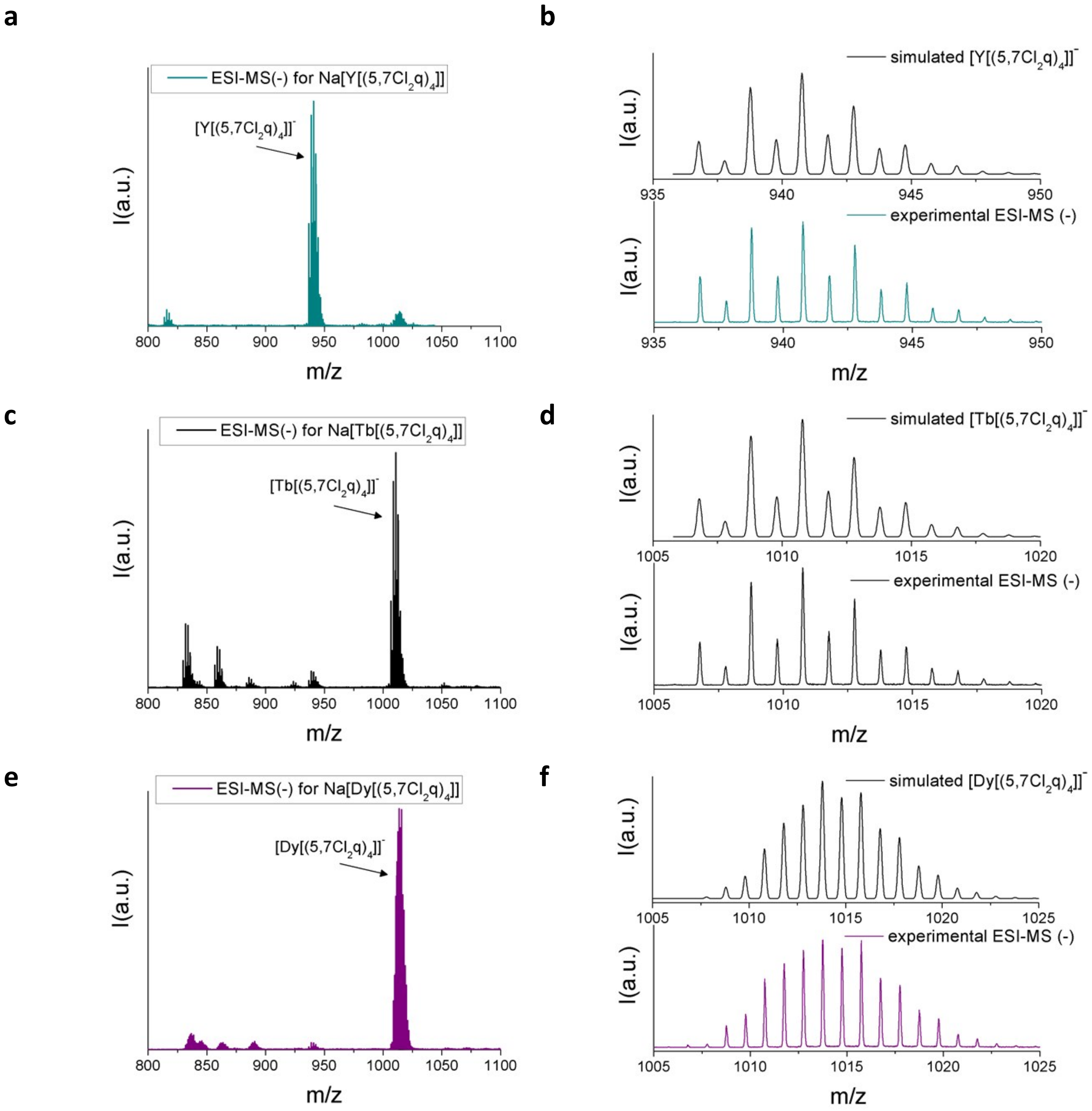

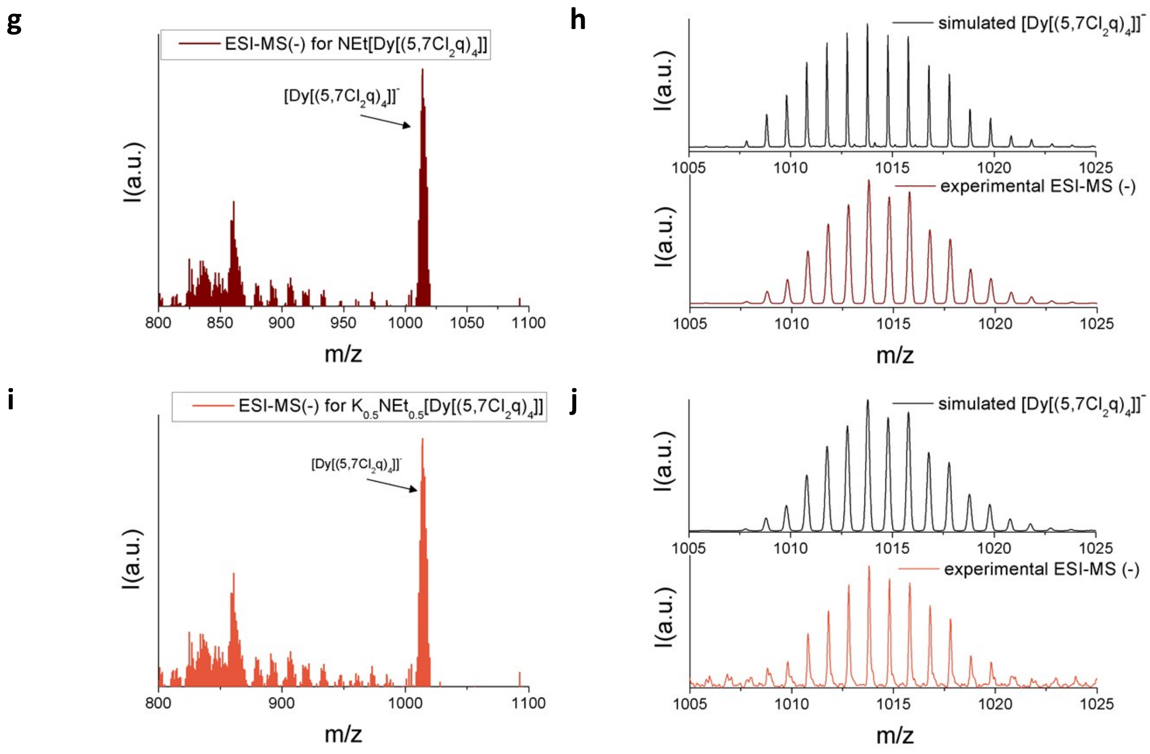

Fig. SI4: *Electrospray ionization mass spectrometry (ESI-MS) for the bulk compounds, a-b* (1), *c-d* (2), *e-f* (3), *g-h* (4) and *i-j* (5), *in negative mode.*

4. Radial Effective Charge (REC) model

Let us start with a caveat: it has been recently pointed out, both experimentally and via theoretical calculations, that spin-vibration coupling are critical for relaxation processes[3–5], meaning the nature of the ground state and the apparent energy barrier are not sufficient conditions to predict SMM behaviour. Despite early successes, currently the necessary theory to fully understand these spin-vibrational-governed relaxation processes is still being developed.

Moreover, even state-of-the-art models for determining the energy level scheme of the magnetic levels, which are much more mature, have important limitations. This has been recently studied with a benchmark study[6] With that being said, one of said state-of-the-art models is the Radial Effective Charge (REC) model, so we apply it here to verify a high-spin ground state, separated by a non-negligible energy from the first excited states as indications for a potential SMM behaviour.

Our calculations start with the crystallographic/non-idealized atomic coordinates of the first coordination sphere. These are introduced as an input for the portable *fortran77* software code SIMPRE[7]. This code parameterizes the electric field effect produced by the surrounding ligands, acting over the central ion, by using the following Crystal Field (CF) Hamiltonian expressed in terms of the Extended Stevens Operators (ESOs)[8,9]:

$$\hat{H}_{cf}(J) = \sum_{k=2,4,6} \sum_{q=-k}^{k} B_k^q O_k^q = \sum_{k=2,4,6} \sum_{q=-k}^{k} a_k (1-\sigma_k) A_k^q \langle r^k \rangle O_k^q \qquad \text{(S1)}$$

where *k* is the order (also called rank or degree) and *q* is the operator range, that varies between *k* and –*k*, of the Stevens operator equivalents O_k^q as defined by Ryabov in terms of the angular momentum operators $J_\pm$ and J_z,[10] where the components $O_k^q(c)$ and $O_k^q(s)$ correspond to the ESOs with q ≥ 0 and q < 0 respectively[10]. Note that all the Stevens CF parameters B_k^q are real, whereas the matrix elements of O_k^q (q < O) are imaginary. a_k are the α, β and γ Stevens coefficients[11] for *k* = 2, 4, 6, respectively, which are tabulated and depend on the number of *f* electrons. σ_k are the Sternheimer shielding parameters[12] of the 4*f* electronic shell, and $\langle r^k \rangle$ are the expectation values of the radius[12].

In SIMPRE, the A_k^q CF parameters are determined by the following relations:

$$A_k^0 = \frac{4\pi}{2k+1}\sum_{i=1}^{N}\frac{Z_i e^2}{R_i^{k+1}} Z_{k0}(\theta_i,\varphi_i)p_{kq} \tag{S2.a}$$

$$A_k^q = \frac{4\pi}{2k+1}\sum_{i=1}^{N}\frac{Z_i e^2}{R_i^{k+1}} Z_{kq}^c(\theta_i,\varphi_i)p_{kq} \tag{S2.b}$$

$$A_k^q = \frac{4\pi}{2k+1}\sum_{i=1}^{N}\frac{Z_i e^2}{R_i^{k+1}} Z_{k|q|}^s(\theta_i,\varphi_i)p_{k|q|} \tag{S2.c}$$

In the REC model[13] the ligand is modeled through an effective point charge situated between the lanthanoid and the coordinated atom at a distance R_i from the magnetic center, which is smaller than the real metal-ligand distance (r_i). To account for the effect of covalent electron sharing, a radial displacement vector (D_r) is defined, in which the polar coordinate r of each coordinated atom is varied, $R_i = r_i - D_r$. The usual procedure is to obtain the D_r parameter of each kind of donor atom from a collective fit of an observable (e.g. energy levels or magnetic properties) for a family of isostructural lanthanide complexes. At the same time, the charge value (Z_i) is scanned in order to achieve the minimum deviation between calculated and experimental data, whereas θ_i and φ_i remain constant. We calculate the effective distances of the coordinated atoms using the following formula for D_r:

$$D_r \approx \left(\frac{N_L}{V_M}\right)\cdot\frac{1}{E_M(E_L - E_M)} \tag{S3}$$

where N_L is the coordination number of the complex ($N_L = 8$), V_M is the valence of the metal ($V_M = 3$), and E_M and E_L are the Pauling electronegativities of the metal ($E_M \approx 1.2$) and the donor atom ($E_L = 3.44$ for oxygen and 3.04 for nitrogen) respectively.

Such relation is an approximation that was obtained by fitting the experimental energy levels of the ground multiplet of the homoleptic families $CsNaYCl_6:Ln^{3+}$ and $CsNaYF_6:Ln^{3+}$, $LiYF_4:Ln^{3+}$ and $LaCl_3:Ln^{3+}$ using the crystal structures and the REC model[13]. The obtained values of *Dr* (N) and *Dr* (O) for the Dy compounds are thus 1.20 Å and 0.98 Å. This strategy allows us to restrict the number of free parameters to 2, i.e. the effective charges of the nitrogen and oxygen atoms, *Zi* (N) = 0.160 and *Zi* (O) = 0.235, which have been obtained by a two-parameter fit of the χT product of NaTbClq and NaDyClq measured under a magnetic field of 0.1 and 1T. In the fitting procedures, we define the relative error *E* as:

$$E = \frac{1}{n}\sum_{i=1}^{n}\frac{\left[\chi_{theo,i} - \chi_{\exp,i}\right]^2}{\left[\chi_{\exp,i}\right]^2} \tag{S4}$$

where χ_{exp} and χ_{theo} are experimental and theoretical magnetic susceptibility, respectively, and n is the number of points.

Subsequently, such REC parameters for both different donor atoms are validated with the correct prediction of the χT product of NEtDyClq and KNEtDyClq, with an excellent agreement with the experimental results (Fig. SI5(b) and SI5(c)). According to this description, the first excited state is found at 126 cm^{-1} (g_z = 19.5), 49 cm^{-1} (g_z = 18.5), 193 cm^{-1} (g_z = 19.4) and 154 cm^{-1} (g_z = 19.2), for NaDyClq, NEtDyClq and KNEtDyClq respectively, with a wave function with a major contribution of M_J = ±15/2 (Fig. SI6(b)) in all cases.

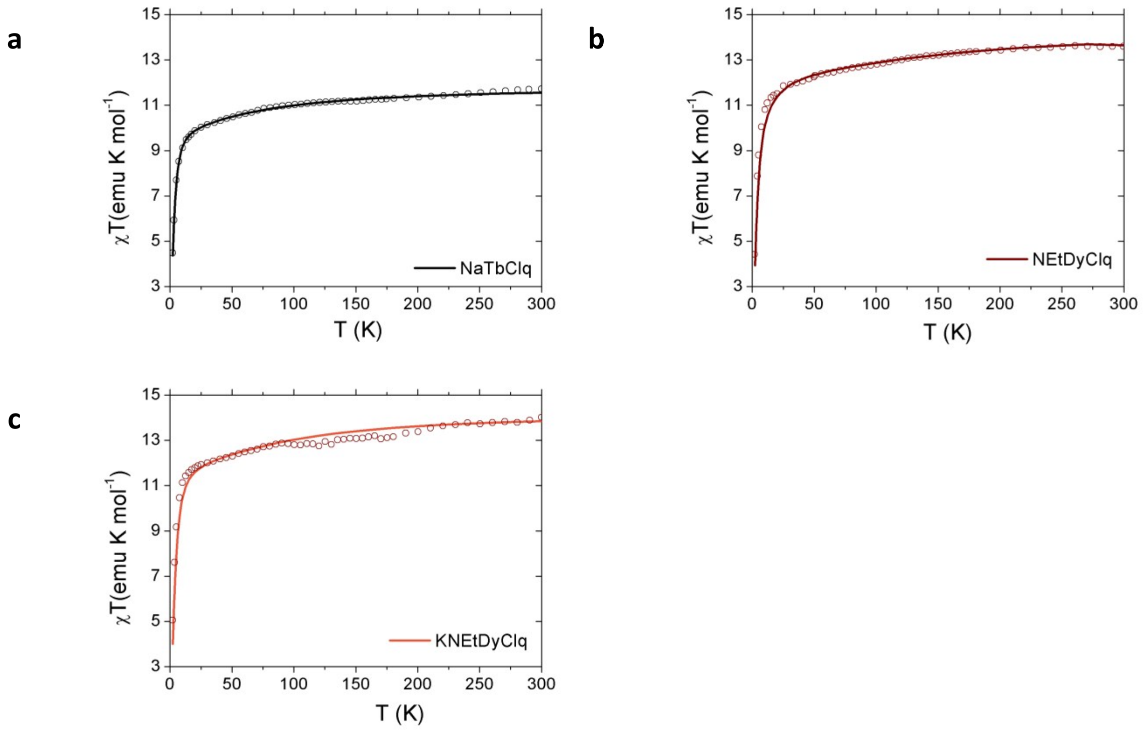

***Fig. SI5:** χT product of a) NaTbClq (**2**), b) NEtDyClq (**4**) and c) KNEtDyClq (**5**) (experimental data in circles) and theoretical fit/prediction from 2 to 300 K at H = 1 T as a solid line.*

The calculated wave functions of the ground doublets, dominated by high-M_J contributions (M_J (Tb) = ±6 or M_J (Dy) = ±15/2) are then compatible with the observed SMM behavior for the four compounds (see Fig. SI6). The different countercations present in each crystallographic structure distort the coordination environment to an extent that is comparable to the differences between two conformers within the same crystal structure. For example, the crystal field effect is slightly larger for (**3**) derivative (about 873 cm^{-1}), compared with (**4**) (800 cm^{-1}) or with the two conformers of (**5**) (841 and 788 for Dy1 and Dy2 respectively). There is no correlation with the averaged distances between the lanthanide and the donor atoms (2.435 Å (**3**), 2.443 Å (**4**), 2.430 Å (**5**) and 2.436 Å (**5´**)), and thus such differences in the crystal field strength are attributed to the small distortions of the chemical structures due to the crystal packing. In contrast with typically negligible distortions caused by temperature[14] the differences in the crystallographic sites, induced partially by these countercations, lead to different energy level schemes, as can be seen in fig. SI6(b). As these variations cannot be easily controlled, there is no immediate connection between countercation and dynamic magnetic properties. In any case, the countercation has no effect on our parameterization of the effective charges, as demonstrated by the good agreement between the predictions of the magnetic data of (**4**) and (**5**) and the experiment (fig. SI5(b) and SI5(c)).

a

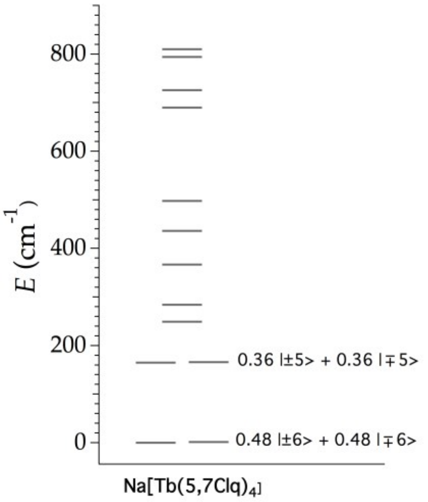

b

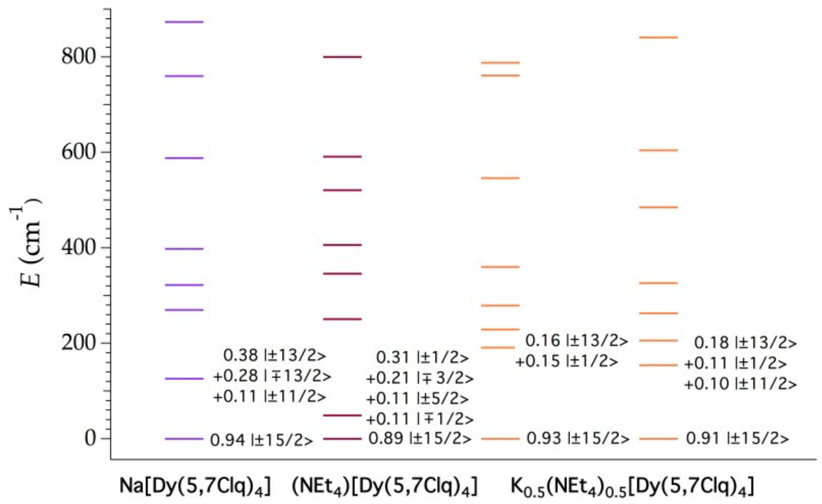

Figure SI6: *Energy level scheme and main M_J contributions to the wave functions of the ground and first excited states of* (2) *NaTbClq (a) and* (3) *NaDyClq,* (4) *NEtDyClq, and* (5) *KNEtDyClq (b) predicted by SIMPRE software.*

5. AC Measurements

The single ion magnet behavior has been experimentally checked by applying an ac magnetic field at different frequencies. Magnetic compounds didn't show frequency dependence at zero dc field (H_{DC}) but they did when a dc field was applied.

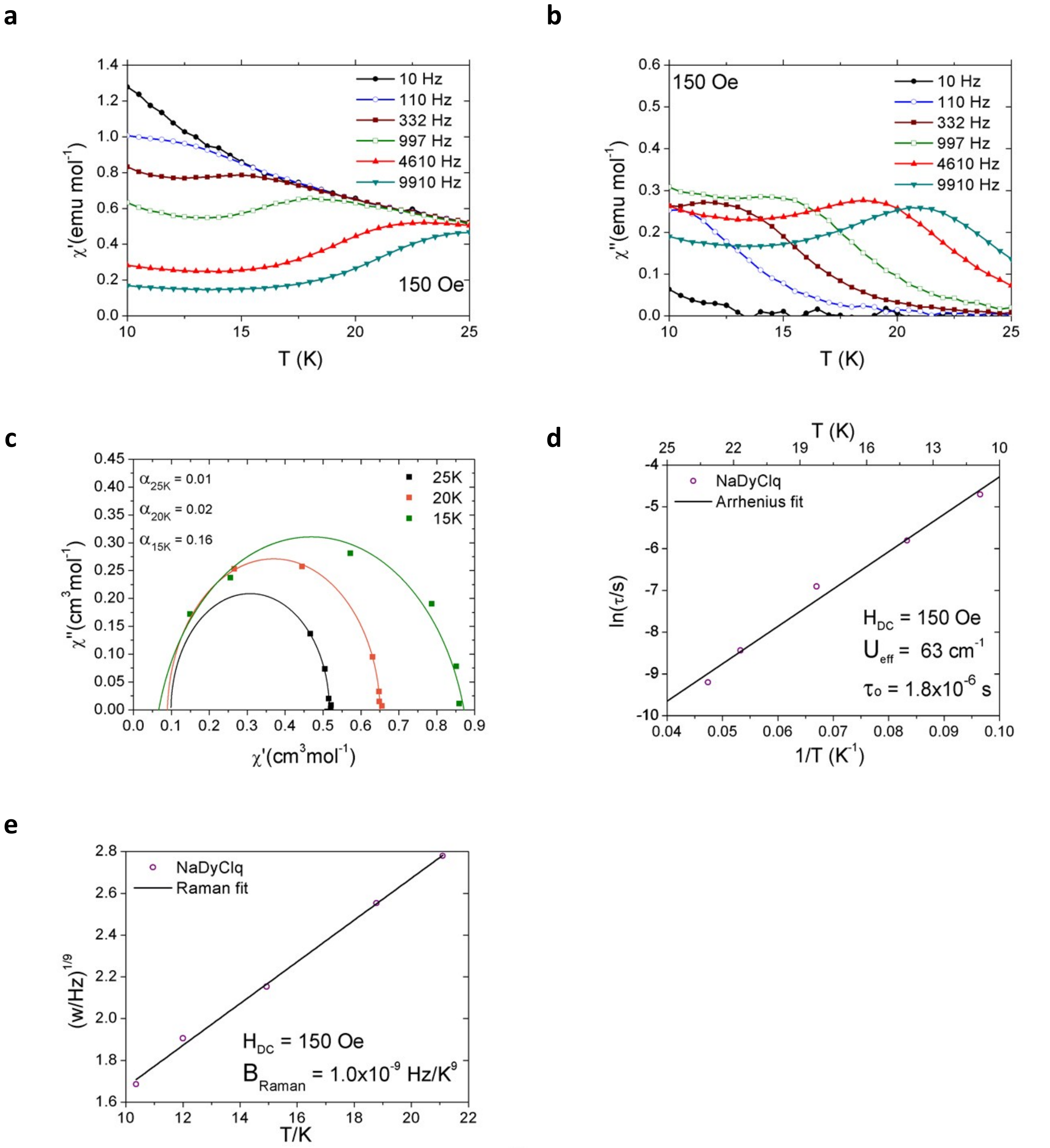

Figure SI7*: Bulk NaDyClq* (3) *ac measurements at 150 Oe. a) Magnetic susceptibility in phase for different frequencies. b) Magnetic susceptibility out of phase. c) Cole-Cole plots at different temperatures with the corresponding α values. The lines in the Cole-Cole plots are fittings to equation SI7. d) Arrhenius fit with an effective energy barrier* U_{eff} *= 63 cm*$^{-1}$*, and a pre-exponential factor* τ_o *= 1.8x10*$^{-6}$ *s. e) Fit to a Raman relaxation mechanism with* B_{Raman} *= 1.0x10*$^{-9}$*HzK*$^{-9}$*.*

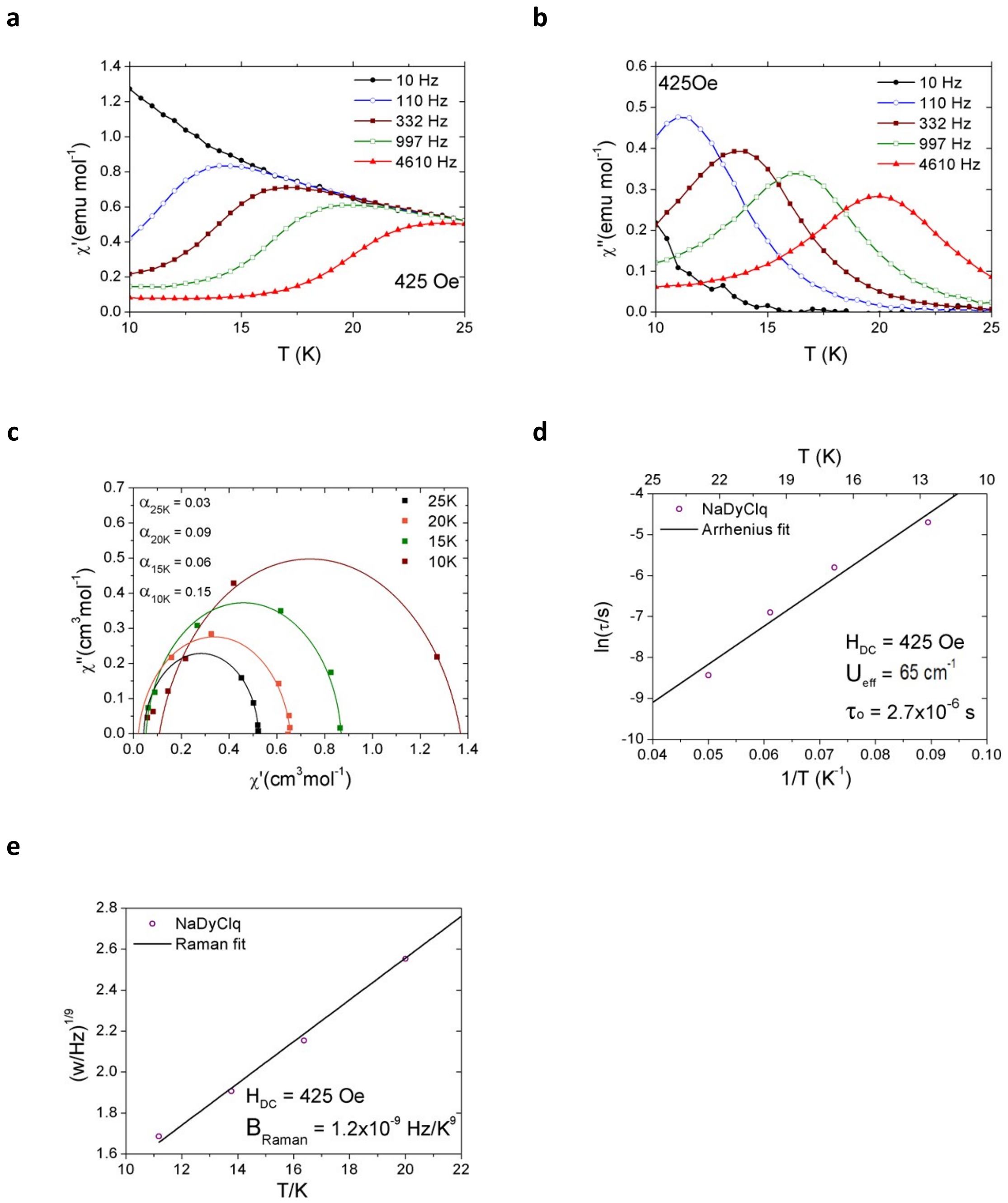

Figure SI8: *Bulk NaDyClq* (3) *ac measurements at 425 Oe. a) Magnetic susceptibility in phase for different frequencies. b) Magnetic susceptibility out of phase. c) Cole-Cole plot at different temperatures with the corresponding α values. The lines in the Cole-Cole plots are fittings to equation SI7. d) Arrhenius fit with an effective energy barrier* U_{eff} *= 65 cm*$^{-1}$*, and a pre-exponential factor* τ_o *= 2.7x10*$^{-6}$ *s. e) Fit to a Raman relaxation mechanism with* B_{Raman} *= 1.2x10*$^{-9}$*HzK*$^{-9}$*.*

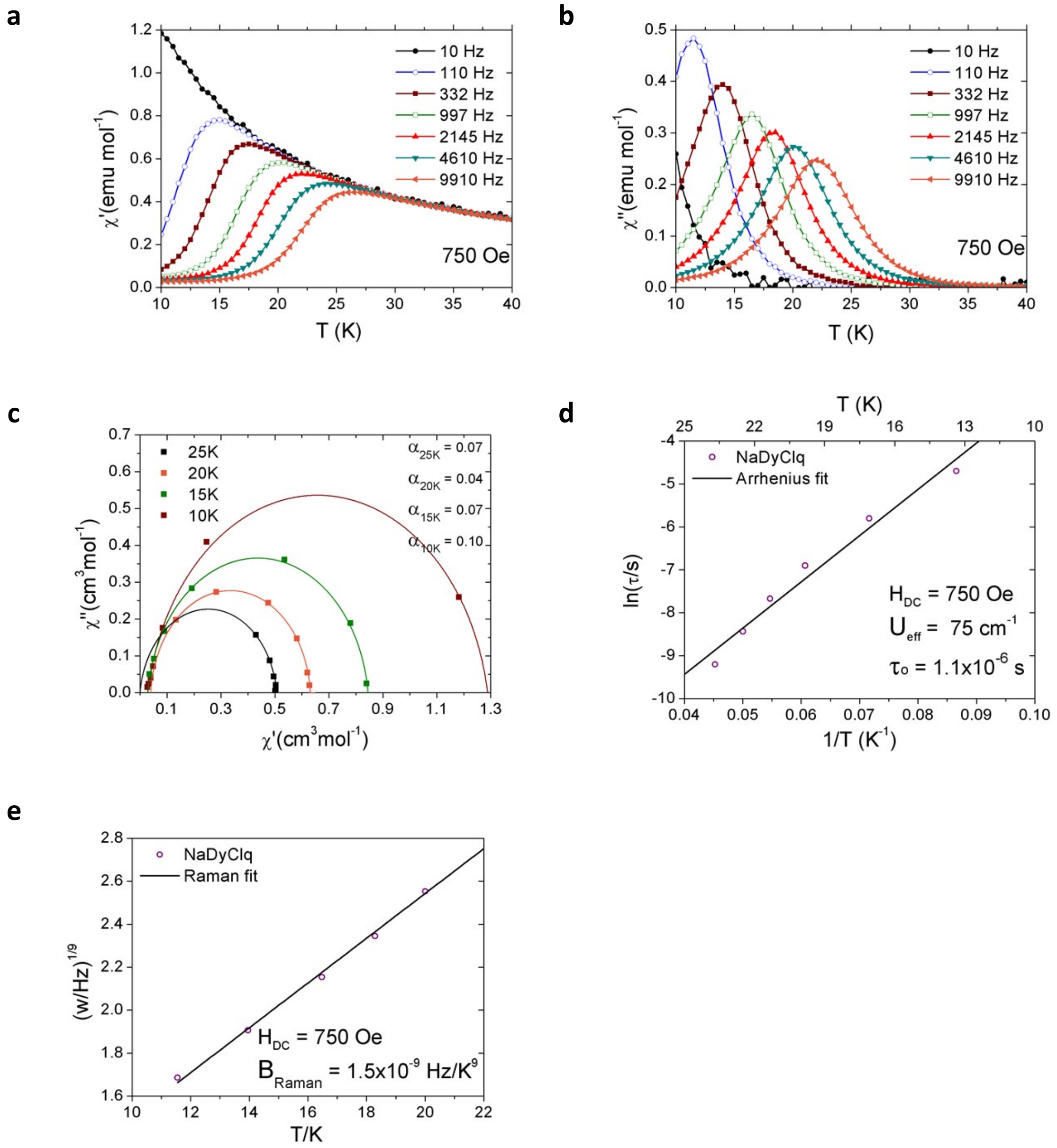

Figure SI9: *Bulk NaDyClq* (3) *ac measurements at 750 Oe. a) Magnetic susceptibility in phase for different frequencies. b) Magnetic susceptibility out of phase. c) Cole-Cole plots at different temperatures with the corresponding α values. The lines in the Cole-Cole plots are fittings to equation SI7. d) Arrhenius fit with an effective energy barrier* $U_{eff} = 75\ cm^{-1}$*, and a pre-exponential factor* $\tau_o = 1.1x10^{-6}$ *s. e) Fit to a Raman relaxation mechanism with* $B_{Raman} = 1.5x10^{-9} HzK^{-9}$.

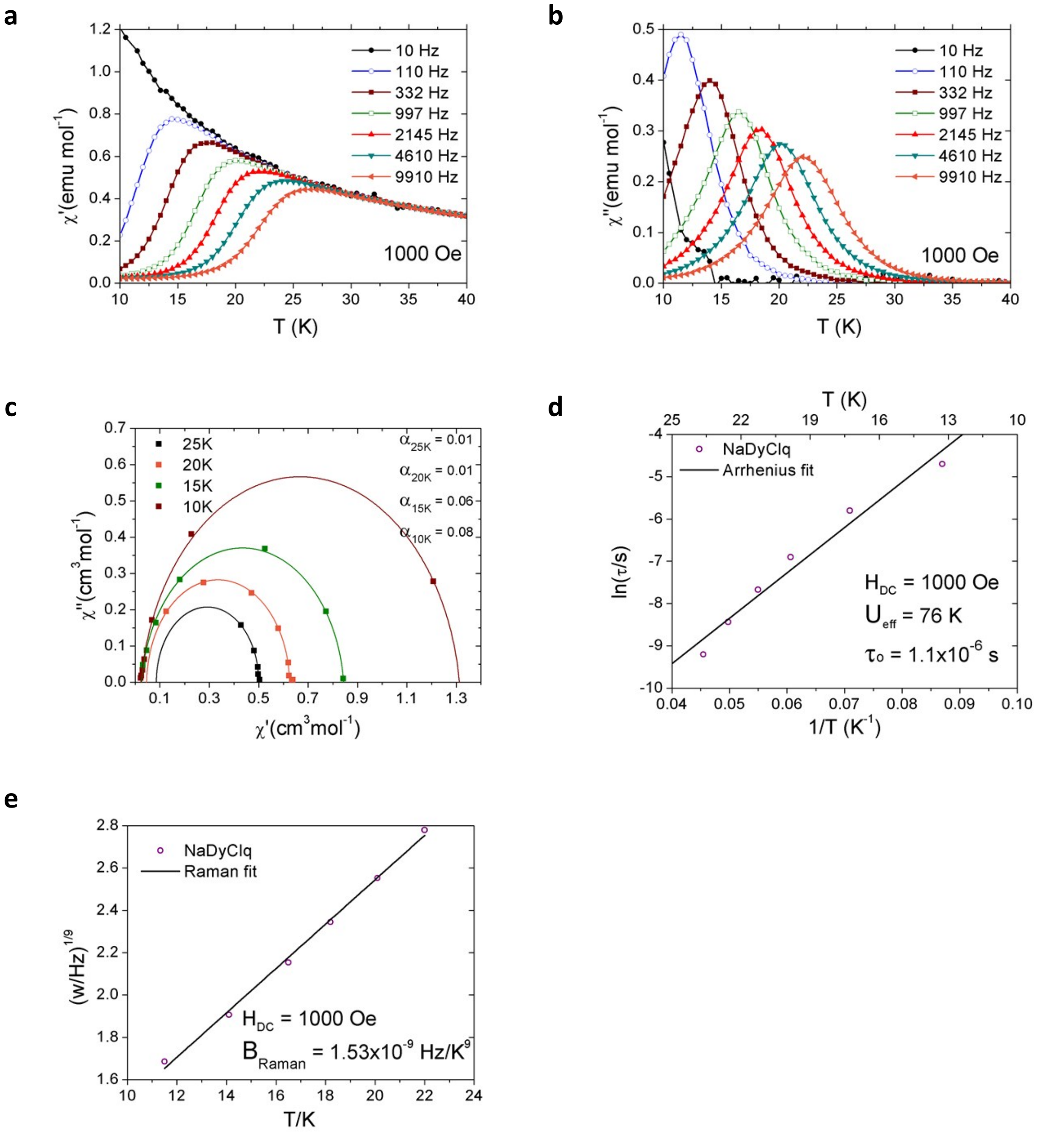

Figure SI10: *Bulk NaDyClq* (**3**) *ac measurements at 1000 Oe. a) Magnetic susceptibility in phase for different frequencies. b) Magnetic susceptibility out of phase. c) Cole-Cole plot at different temperatures with the corresponding α values. The lines in the Cole-Cole plots are fittings to equation SI7. d) Arrhenius fit with an effective energy barrier* $U_{eff} = 76\ cm^{-1}$*, and a pre-exponential factor* $\tau_o = 1.1x10^{-6}$ *s. e) Fit to a Raman relaxation mechanism with* $B_{Raman} = 1.53x10^{-9} HzK^{-9}$.

a b
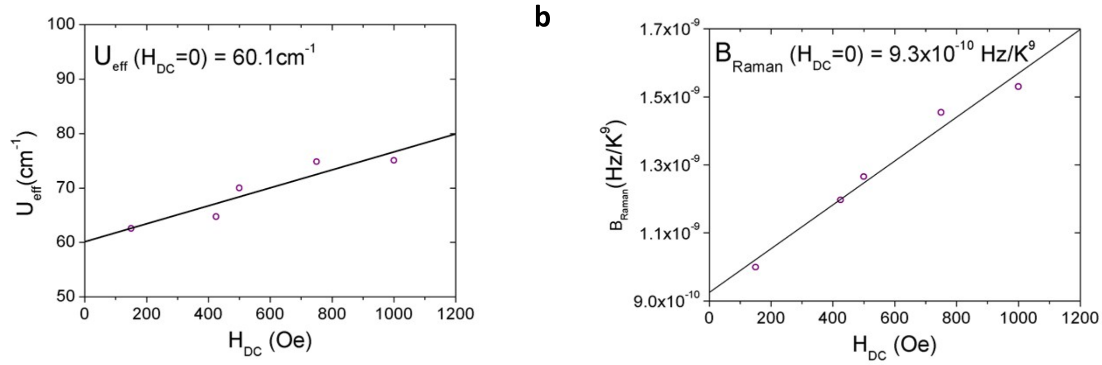

Figure SI11: *a) Extrapolation of the effective energy barrier (U_{eff} = 60.1 cm^{-1}) at zero dc magnetic field for bulk NaDyClq* (**3**)*. b) Extrapolation of C at zero dc field with B_{Raman} = $9.3x10^{--10}HzK^{-9}$ for the same compound.*

$$\tau = \tau_o e^{-\frac{U_{eff}}{k_B T}}$$

Equation SI5: *Arrhenius equation.*

$$\tau^{-1} = B_{Raman} T^n$$

Equation SI6: *Raman relaxation equation.*

$$\chi''(\chi') = \frac{-\chi_T - \chi_S}{2\tan\left((1-\alpha)\frac{\pi}{2}\right)} + \sqrt{(\chi' - \chi_S)(\chi_T - \chi')\frac{(\chi_T - \chi_S)^2}{2\tan\left((1-\alpha)\frac{\pi}{2}\right)}}$$

Equation SI7: *Cole-Cole equation where α (0 < α < 1) is related to the number of relaxation mechanisms, χ_T is the isothermal susceptibility and χ_S is the adiabatic susceptibility.*

Table SI8: *Coefficient of determination, R^2, of the fits to the Raman and Orbach terms showing a best fit for the Raman relaxation in all cases for the compound* (**3**)*.*

H(Oe)	$R^2(\tau^{-1} = CT^{1/9})$	$R^2(\tau^{-1} = \tau_o^{-1}exp(-U_{eff}/kT))$
150	0.997	0.983
425	0.996	0.957
500	0.995	0.954
750	0.997	0.966
1000	0.996	0.985

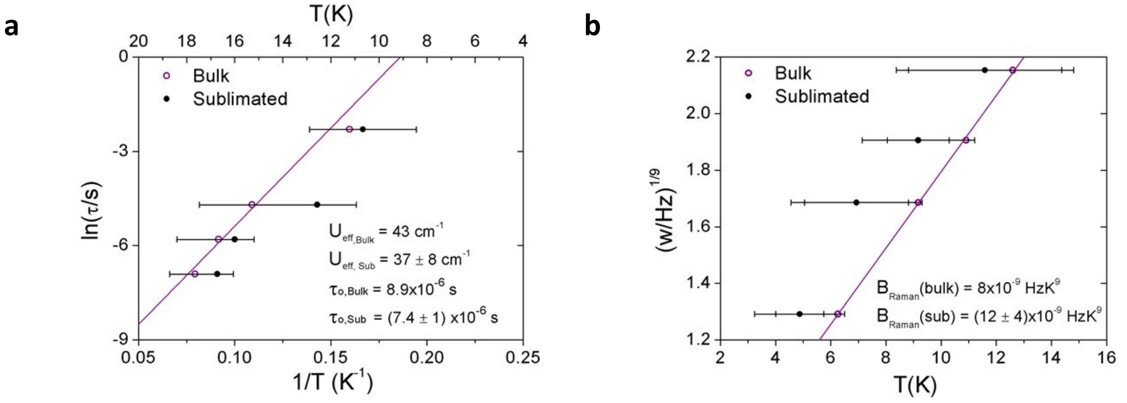

Figure SI12: *a) Comparation of the Raman mechanism values for NaDyClq* (**3**) *as bulk and sublimated material measured in a SQUID magnetometer. For the linear fit to equation SI5, only the points at high temperature (13 – 20 K) are taken into account. b) Equivalent Raman mechanism fits.*

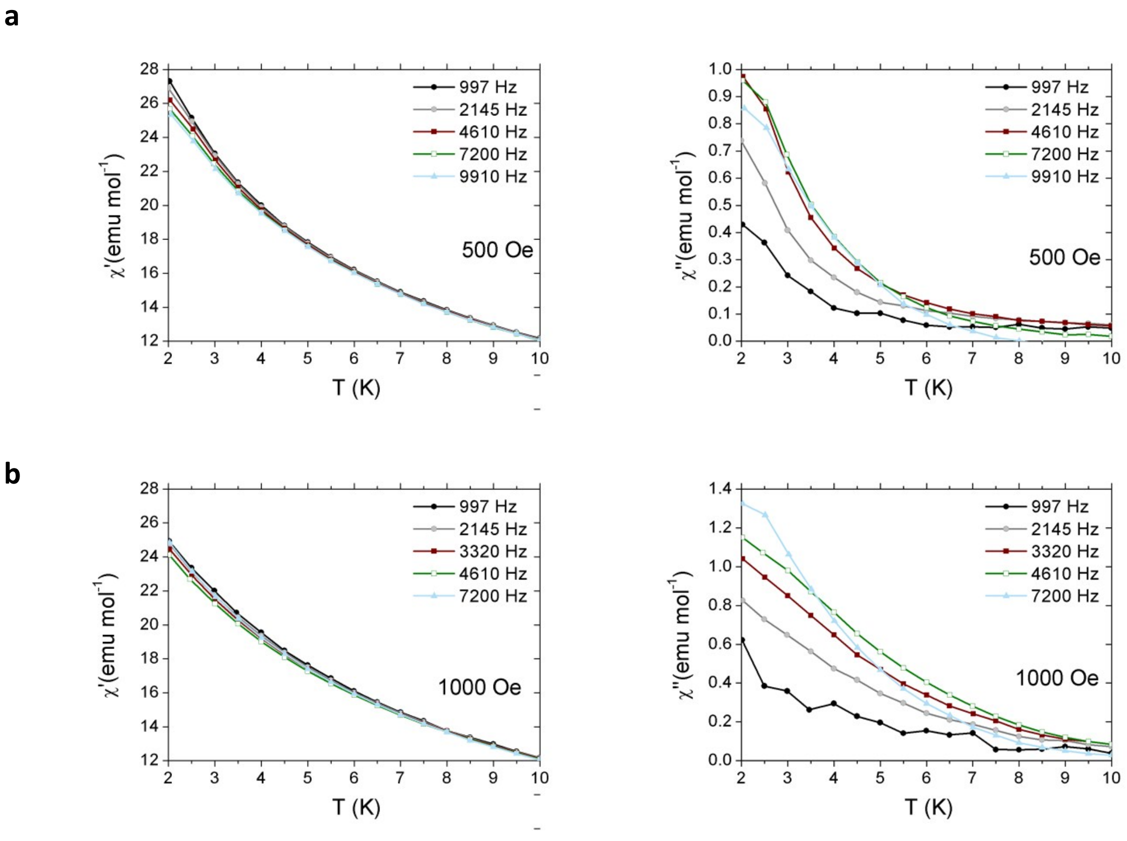

Figure SI13: *Magnetic susceptibilities in phase (χ', left) and out of phase (χ'', right) at a) H_{DC} = 500 Oe and b) H_{DC} = 2000 Oe of bulk NaTbClq* (**2**).

a

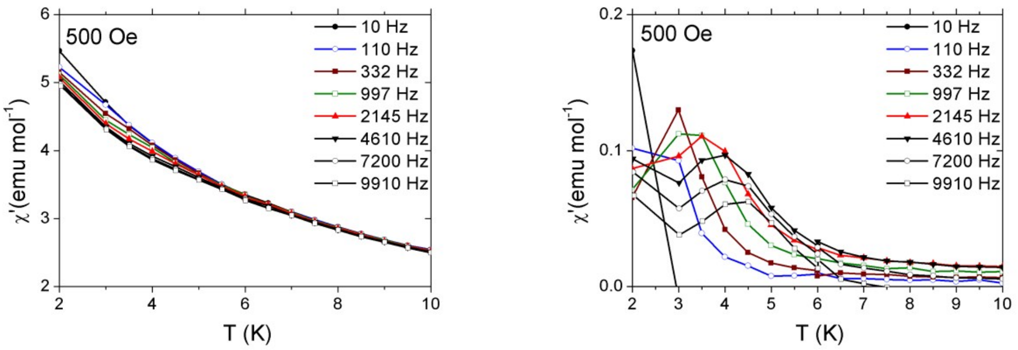

b

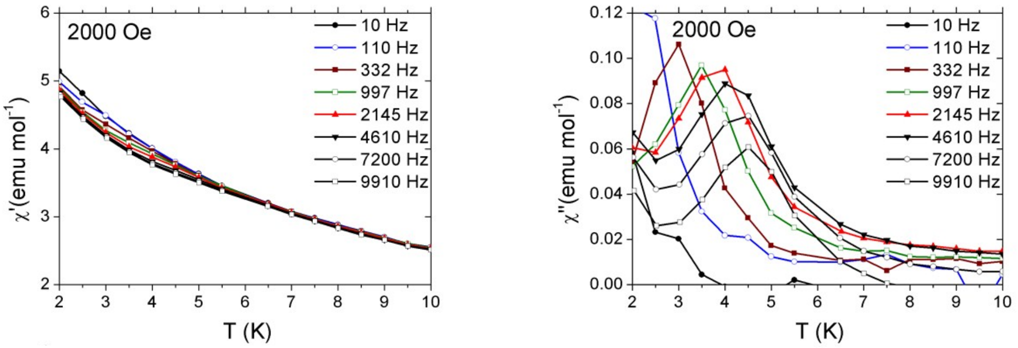

Figure SI14: *Magnetic susceptibilities in phase (χ', left) and out of phase (χ'', right) at a) H_{DC} = 500 Oe and b) H_{DC} = 2000 Oe of bulk NEtDyClq (**4**).*

6. Film Characterization

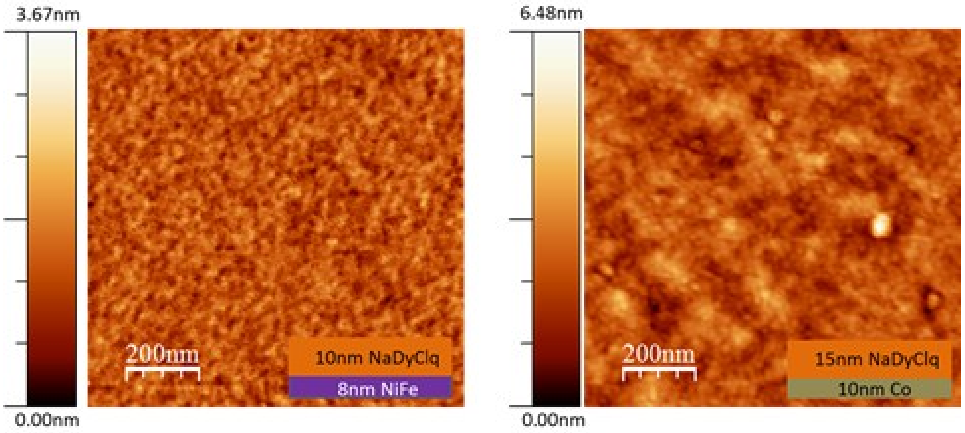

Figure SI15: *AFM topography images of NaDyClq* (**3**) *molecular layers grown on NiFe (left) and Co (right) substrates.*

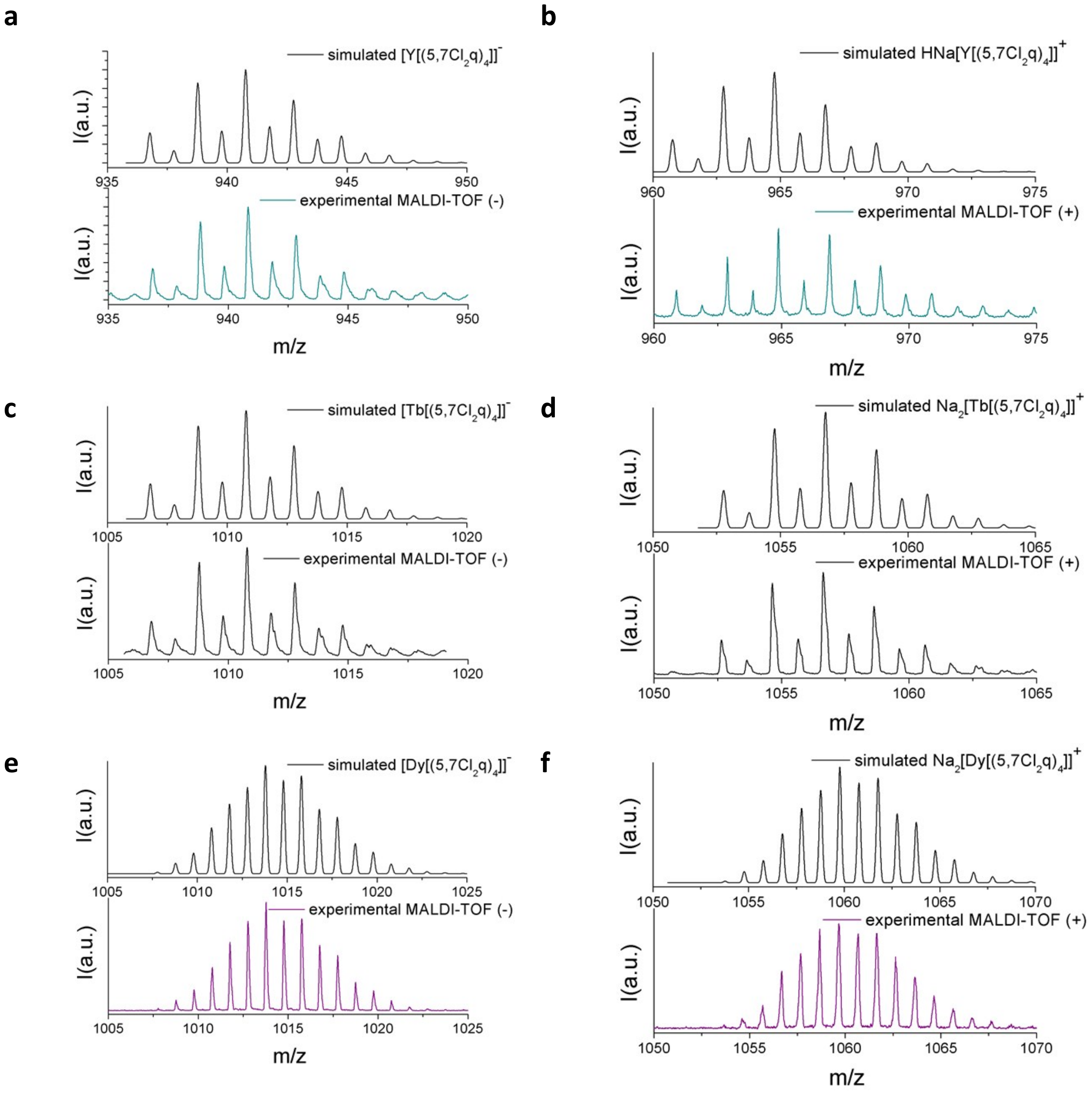

Figure SI16: *Negative and positive modes MALDI-TOF for films of a) NaYClq* (**1**)*, b)*

NaTbClq (**2**) *and c) NaDyClq* (**3**). *A pattern was not found in films of NEtDyClq* (**4**) *and KNEtDyClq* (**5**).

a

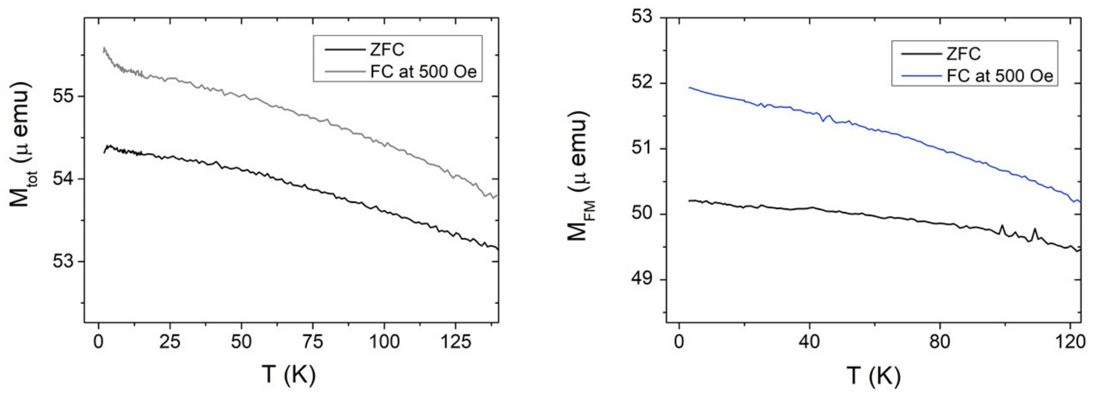

Figure SI17: a) Temperature-dependent magnetization of NiFe (10nm)/NaTbClq where a 1/T behavior is observed in the FC curve whereas a blocking (cusp) appears in the ZFC curve. **b)** Temperature-dependent magnetization of NiFe (10 nm). M_{FM} has been subtracted in Figures 5b-d and in some cases scaled to M_{tot} at low temperatures before subtraction, as slight variation in the thickness yield different absolute magnetization values. recalcar que el NiFe no tiene 1/T behaviour en el fc ni cusp en el zfc (blocking).

References

(1) Aly, H. F.; Abdel Kerim, F. M.; Kandil, A. T. I.R. Spectra of Lanthanide 8-Hydroxyquinoline Complexes. *J. Inorg. Nucl. Chem.* **1971**, *33*, 4340–4344.

(2) Nervik, W. E.; Magee, R. J.; Freiser, H.; Friedel, R.; Hillard, L. E.; Johnson, W. D. I.R. Spectra of Lanthanide 8-Hydroxyquinoline Complexes. **1971**, *33*, 4340–4344.

(3) Lunghi, A.; Totti, F.; Sessoli, R.; Sanvito, S. The Role of Anharmonic Phonons in under-Barrier Spin Relaxation of Single Molecule Magnets. *Nat. Commun.* **2017**, *8*, 14620.

(4) Escalera-Moreno, L.; Suaud, N.; Gaita-Ariño, A.; Coronado, E. Determining Key Local Vibrations in the Relaxation of Molecular Spin Qubits and Single-Molecule Magnets. *J. Phys. Chem. Lett.* **2017**, *8*, 1695–1700.

(5) Chilton, N. F.; Deacon, G. B.; Gazukin, O.; Junk, P. C.; Kersting, B.; Langley, S. K.; Moubaraki, B.; Murray, K. S.; Schleife, F.; Shome, M.; *et al.* Structure, Magnetic Behavior, and Anisotropy of Homoleptic Trinuclear Lanthanoid 8-Quinolinolate Complexes. *Inorg. Chem.* **2014**, *53*, 2528–2534.

(6) Baldoví, J. J.; Duan, Y.; Morales, R.; Gaita-Ariño, A.; Ruiz, E.; Coronado, E. Rational Design of Lanthanoid Single-Ion Magnets: Predictive Power of the Theoretical Models. *Chem. - A Eur. J.* **2016**, *22*, 13532–13539.

(7) Baldoví, J. J.; Cardona-Serra, S.; Clemente-Juan, J. M.; Coronado, E.; Gaita-Ariño, A.; Palii, A. SIMPRE: A Software Package to Calculate Crystal Field Parameters, Energy Levels, and Magnetic Properties on Mononuclear Lanthanoid Complexes Based on Charge Distributions. *J. Comput. Chem.* **2013**, *34*, 1961–1967.

(8) Rudowicz, C.; Chung, C. Y. The Generalization of the Extended Stevens Operators to Higher Ranks and Spins, and a Systematic Review of the Tables of the Tensor Operators and Their Matrix Elements. *J. Phys. Condens. Matter* **2004**, *16*, 5825–5847.

(9) Rudowicz, C. Transformation Relations for the Conventional Ok Q and Normalised O'k Q Stevens Operator Equivalents with k=1 to 6 and -K≤q≤k. *J. Phys. C Solid State Phys* **1985**, *18*, 1415–1430.

(10) Ryabov, I. D. On the Generation of Operator Equivalents and the Calculation of Their Matrix Elements. *J. Magn. Reson.* **1999**, *140*, 141–145.

(11) Stevens, K. W. H. Matrix Elements and Operator Equivalents Connected with the Magnetic Properties of Rare Earth Ions. *Proc. Phys. Soc. Sect. A* **2002**, *65*, 209–215.

(12) Edvardsson, S.; Klintenberg, M. Role of the Electrostatic Model in Calculating Rare-Earth Crystal-Field Parameters. *J. Alloys Compd.* **1998**, *275–277*, 230–233.

(13) Baldoví, J. J.; Borrás-Almenar, J. J.; Clemente-Juan, J. M.; Coronado, E.; Gaita-Ariño, A. Modeling the Properties of Lanthanoid Single-Ion Magnets Using an Effective Point-Charge Approach. *Dalton Trans.* **2012**, *41*, 13705.

(14) Qian, K.; Baldoví, J. J.; Jiang, S.-D.; Gaita-Ariño, A.; Zhang, Y.-Q.; Overgaard, J.; Wang, B.-W.; Coronado, E.; Gao, S. Does the Thermal Evolution of Molecular Structures Critically Affect the Magnetic Anisotropy? *Chem. Sci.* **2015**, *6*, 4587–4593.
